# Bioorthogonally activatable cyanine dye with torsion-induced disaggregation for in vivo tumor imaging

**DOI:** 10.1038/s41467-022-31136-3

**Published:** 2022-06-18

**Authors:** Xianghan Zhang, Jingkai Gao, Yingdi Tang, Jie Yu, Si Si Liew, Chaoqiang Qiao, Yutian Cao, Guohuan Liu, Hongyu Fan, Yuqiong Xia, Jie Tian, Kanyi Pu, Zhongliang Wang

**Affiliations:** 1 Engineering Research Center of Molecular & Neuroimaging, Ministry of Education, School of Life Science and Technology, Xidian University, Xi’an, Shaanxi 710126 China; 2 Academy of Advanced Interdisciplinary Research, Xidian University, Xi’an, Shaanxi 710071 China; 3 School of Chemical and Biomedical Engineering, Nanyang Technological University, 70 Nanyang Drive, Singapore, 637457 Singapore; 4 Beijing Advanced Innovation Center for Big Data-Based Precision Medicine, School of Medicine, Beihang University, Beijing, 100191 China

**Keywords:** Cancer imaging, Fluorescent probes, Imaging studies, Chemical tools

## Abstract

Advancement of bioorthogonal chemistry in molecular optical imaging lies in expanding the repertoire of fluorophores that can undergo fluorescence signal changes upon bioorthogonal ligation. However, most available bioorthogonally activatable fluorophores only emit shallow tissue-penetrating visible light via an intramolecular charge transfer mechanism. Herein, we report a serendipitous “torsion-induced disaggregation (TIDA)” phenomenon in the design of near-infrared (NIR) tetrazine (Tz)-based cyanine probe. The TIDA of the cyanine is triggered upon Tz-transcyclooctene ligation, converting its heptamethine chain from *S-trans* to *S-cis* conformation. Thus, after bioorthogonal reaction, the tendency of the resulting cyanine towards aggregation is reduced, leading to TIDA-induced fluorescence enhancement response. This Tz-cyanine probe sensitively delineates the tumor in living mice as early as 5 min post intravenous injection. As such, this work discovers a design mechanism for the construction of bioorthogonally activatable NIR fluorophores and opens up opportunities to further exploit bioorthogonal chemistry in in vivo imaging.

## Abstract (web summary)

Expanding the responsive dyes repertoire is currently a developing field in biorthogonal chemistry. In this article, the authors develop fluorophores that turn on their near-infrared fluorescence upon biorthogonal reaction based on a “torsion-induced disaggregation” approach, allowing for sensitive in vivo imaging of tumors.

## Introduction

Near-infrared (NIR) fluorescence imaging has contributed significantly to improving therapeutic outcomes in cancer diagnosis, image-guided surgery, and therapeutic evaluation by real-time visualization^1–8^. NIR fluorescent probes offer deeper light penetration and lesser tissue autofluorescence, giving rise to higher signal-to-background ratios for in vivo imaging^9–14^. Traditional NIR imaging probes target sites of interest via selective binding to biomolecules overexpressed in diseases^10^. However, nonspecific background signals are inevitable because of passive accumulation in normal tissues. In contrast, the signals of smart NIR optical probes are turned on only upon reaction with chemical or enzymatic triggers, enabling disease detection and monitoring of biomarker levels with greater specificity and sensitivity^15–21^. Bioorthogonal chemistry has emerged as a promising tool for designing smart NIR optical probes for sensitive in vivo imaging due to high selectivity, rapid reaction rates, and good biocompatibility^22–25^. In recent years, bioorthogonal chemistry has seen widespread applications in optical imaging such as visualizing dynamic processes of endogenous molecules (i.e., enzymes, lipids, RNA, etc.)^23,26–29^, pre-targeted imaging of disease biomarkers^30–33^, in situ metabolic labeling and imaging and enabling the assembly of molecular imaging agents in vivo for theranostics^34–39^.

**Fig. 1:**
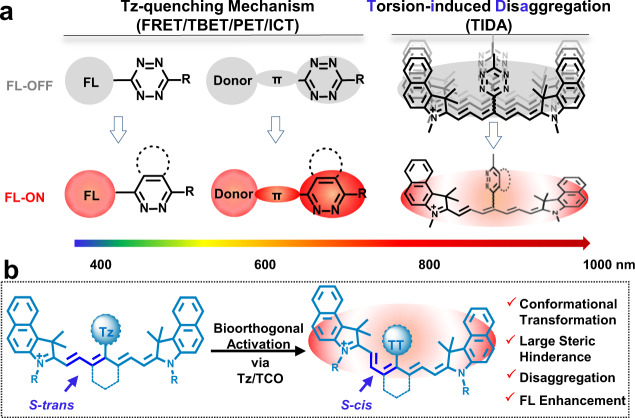
Strategies for Tz-based bioorthogonally activatable probes. Tz-quenching mechanism (**a**) and the torsion-induced disaggregation (TIDA) for activatable NIR Tz-cyanine in our work (**b**).

Further advancement of bioorthogonal chemistry in molecular optical imaging lies in expanding the repertoire of substrates/fluorophores that can undergo fluorescence signal changes upon bioorthogonal ligation. Till date, there remains very few molecular scaffolds in the bioorthogonal chemistry toolbox that can do so. One rare example is the inverse-electron demand Diels–Alder (IEDDA) reaction between tetrazine (Tz) and dienophiles, which occurs with rapid kinetics to ensure efficient ligation even at low concentrations in complex biological systems^40–45^. As fluorophores with direct conjugation of Tz to the electronic structures often have through-bond energy transfer (TBET), förster resonance energy transfer (FRET) or photoinduced electron transfer (PET), Tz-conjugated fluorophores have intrinsically quenched fluorescence^46–51^. After bioorthogonal ligation via IEDDA reaction, changes in the structure of the fluorophores eliminate the quenching effect, leading to fluorescence enhancement response^52,53^. However, most of these fluorophores, such as coumarins, boron-dipyrromethene dyes, xanthenes, silicon-rhodamines and phenoxazines, only emit shallow tissue-penetrating visible light (Fig. 1)^47,48,54–58^. Only few Tz-based NIR fluorescence probes have been developed using intramolecular charge transfer (ICT)^59^, whereby a new conjugated donor–π–acceptor electron system forms upon Tz-dienophile ligation, resulting in fluorescence enhancement. However, potential issues of current NIR Tz-based fluorescence probes include complex synthesis and in vivo autofluorescence. Thus, developing bioorthogonally activatable NIR fluorescence probes remains a field in its infancy.

In this work, we report a serendipitous “torsion-induced disaggregation (TIDA)” phenomenon in the design of our bioorthogonally activatable NIR cyanine probes. Unlike previous work utilizing Tz as a quencher^60^, the Tz moiety is introduced on the meso-position of the heptamethine chain (Fig. 1). Upon bioorthogonal ligation with transcyclooctene (TCO), the introduction of a bulky moiety causes a perturbation in the planarity of the fluorophore. The increase in torsion angle reduces both π-electron delocalization and π–π stacking within the conjugated system, leading to disaggregation of the fluorophores. As such, this TIDA phenomenon gives rise to a fluorescence enhancement response after bioorthogonal reaction. Nitrogen-substituents are incorporated in the fluorophore via amino-Tz to enlarge the Stokes shifts, minimizing in vivo autofluorescence. Such an approach presents less complication in the organic synthesis and could at the same time, achieve highly sensitive imaging both in vitro and in vivo. In addition, PEG-modification is incorporated into the design of the cyanine probe to enhance blood circulation time, promote uptake in tumor tissue while retaining good clearance property. When applied in 4T1 tumor-bearing mice model for in vivo imaging, the successful bioorthogonal activation of the probe affords good sensitivity and specificity in tumor detection. More importantly, this work opens up a vista in employing TIDA as a strategy for the design of bioorthogonally activatable NIR probes.

## Results

### Design and photochemical properties

The majority of NIR probes suitable for in vivo imaging reported thus far are cyanine derivatives. However, their fluorescence suffers from an intrinsic drawback due to aggregation-caused quenching. Commercially available NIR cyanine dyes, such as indocyanine green (ICG) and IR780, are known to show concentration-dependent quenching at concentrations greater than 1 μM (Supplementary Fig. 1). In the case of ICG, fluorescence loss can reach up to 80% at 4 μM. For cyclohexylamine-substituted cyanine, CyP7-CH, 55% fluorescence loss was observed at 4 μM (Supplementary Fig. 2). While fluorescence is favored in organic fluorophores with large conjugated and rigid planar systems, such structures are also prone to aggregation due to π–π stacking. As such, we hypothesized that introducing steric hindrance to the cyanine-based fluorophores would disrupt π–π stacking, thus presenting a simple and effective way to achieve favorable fluorescence properties for various bioimaging applications. Herein, we report a bioorthogonally activatable NIR cyanine-based probe for in vivo tumor imaging via TIDA.

**Fig. 2:**
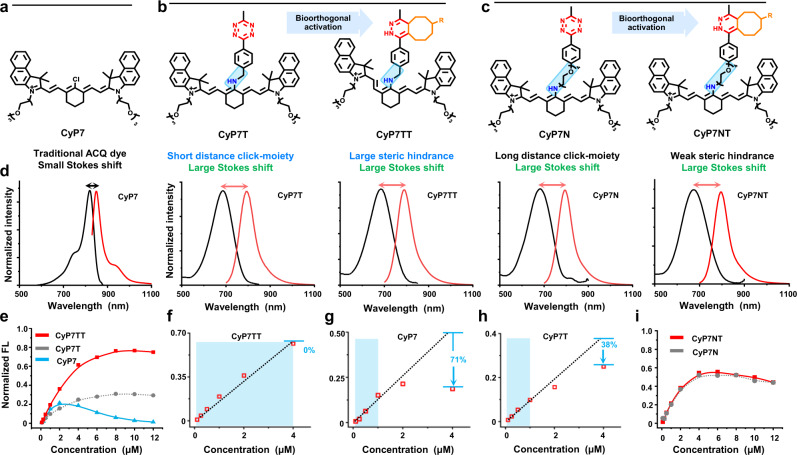
Molecular design and spectra properties of cyanines. Chemical structures of **a** CyP7, **b** bioorthogonal-activated cyanines, and **c** control cyanines. Bioorthogonal-moiety Tz (red color group) with methyl (shorter) (CyP7T) or PEG3 (longer) (CyP7N) distances from substrate structures. CyP7TT/CyP7NT activated by CyP7T/CyP7N and TCO via IEDDA reaction. **d** Normalized absorption (black)/emission (red) spectra of dyes. **e** Fluorescence intensity at the maximum emission spectra with different concentrations of cyanines. **f**–**h** Fluorescence loss with concentrations. Dotted lines represent ideal linear relationship between fluorescence intensity and concentration, indicating non-quenching in blue range. **i** ACQ effect for control group (CyP7NT formed by CyP7N and TCO).

To endow the probe with long half-life in blood circulation, short hydrophilic polyethylene glycol (PEG) chains were introduced to the heterocyclic rings of NIR-benzoindole heptamethine cyanine, yielding CyP7 (Fig. 2a). Next, the Tz moiety was incorporated on CyP7 via nucleophilic substitution of the chloro atom with amino-Tz to produce Tz-Cyanines, CyP7T (Fig. 2b), and CyP7N (Fig. 2c). Following which, Tz-TCO ligation was carried out on the Tz-Cyanines (CyP7T or CyP7N) to generate CyP7TT and CyP7NT respectively. Successful Tz-TCO ligation was confirmed by HRMS, ^1^H NMR and ^13^C NMR analysis of the resulting conjugates, CyP7TT and CyP7NT (Supplementary Figs. 3–26). The second-order rate constant of Tz-TCO ligation was determined to be 5.5 ± 0.9 M^−1^ s^−1^ for the reaction between CyP7T with TCO (Supplementary Fig. 27). After introducing a nitrogen atom to the meso-position of the heptamethine chain, N-cyanines (CyP7-CH, CyP7T, CyP7TT, CyP7N, and CyP7NT) demonstrated large Stokes shifts of 94‒112 nm, compared to CyP7’s shift of 29 nm (Fig. 2d and Supplementary Table 1). Gratifyingly, upon Tz-TCO ligation of CyP7T, the conjugate CyP7TT exhibited 2.5-fold higher fluorescence and concentration-dependent fluorescence enhancement up to 8 μM (Fig. 2e). Notably, CyP7TT showed almost no fluorescence loss even at 4 μM (Fig. 2f). In contrast, strong fluorescence quenching was observed for both CyP7 (71%) and CyP7T (38%) at 4 μM (Fig. 2g–h). We deduced that the introduction of TCO moiety via Tz-TCO ligation may have disrupted π–π stacking between the fluorophores, leading to the disaggregation of CyP7TT even at high concentrations, which is different from Tz-quenching mechanism^61^. To further substantiate this hypothesis, negative control probe CyP7N (Fig. 2c), with longer PEG linker between the Tz moiety and heptamethine chain was designed to mitigate the steric hindrance caused by introduction of TCO moiety on the fluorophore. As expected, we observed almost no difference in fluorescence quenching between CyP7N and CyP7NT (Fig. 2i). This confirmed our hypothesis that weaker steric hindrance gave rise to minimal π–π stacking disruption and hence, lesser disaggregation in CyP7NT compared to CyP7TT (Fig. 2c). To elucidate the mechanism behind this phenomenon, theoretical calculations, NMR studies, and modeling simulations were next carried out.

### Theoretical calculations

**Fig. 3:**
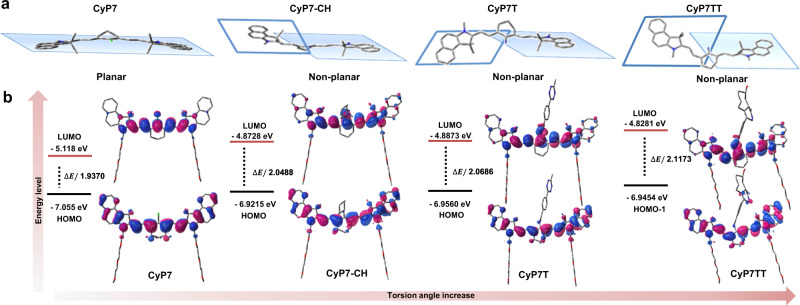
Mechanism for TIDA effect under theoretical calculations. **a** Representative molecular structures and conformations of CyP7, CyP7-CH, CyP7T, and CyP7TT cyanines optimized at B3LYP/6-31G* level (hydrogen atoms, N-substituents on benzoindole rings and meso-amino-substituents are neglected for clear vision). **b** HOMO/HOMO-1/LUMO orbitals and energies with the largest oscillator strengths for CyP7, CyP7-CH, CyP7T, and CyP7TT (hydrogen atoms are neglected).

To investigate the above dequenching phenomenon observed in CyP7TT after Tz-TCO ligation, density functional theory calculations were performed to determine the lowest energy conformations, frontier molecular orbitals as well as electronic transition energies of CyP7, CyP7-CH, CyP7T, and CyP7TT. As shown in Fig. 3a and Supplementary Fig. 28, CyP7 has a highly rigid and planar π-conjugated system with torsion angle of 0.009°. However, nitrogen-substituted cyanines, CyP7-CH and CyP7T, were out-of-plane with torsion angles of 28.602° and 33.149°, respectively. After Tz-TCO ligation, CyP7TT showed the largest torsion angle of 44.354°. For the negative control groups CyP7N/CyP7NT, as expected, almost no difference was observed for their torsion angles (34.967° and 34.664°) after bioorthogonal reaction (Supplementary Fig. 28). Encouraged by the above results showing a relationship between non-planarity of the molecule and fluorescence emission, the excited-state property of the cyanine dyes was also determined. Figure 3b and Supplementary Table 2 show that S_0_-S_1_ excitations with the largest oscillator strength originated from ICT via HOMO/LUMO for CyP7. The twisted intramolecular charge transfer (TICT) was generated via HOMO/LUMO and HOMO-1/LUMO for CyP7-CH, CyP7T and CyP7TT, respectively. The HOMO and LUMO energy levels of the cyanine dyes increased with increasing torsion angles. Meanwhile, the HOMO-LUMO energy gaps increased in the order CyP7TT > CyP7T > CyP7-CH > CyP7, suggesting that the increase in torsion angle contributed to enlarging the HOMO–LUMO energy gaps, resulting in reduced π-electron delocalization within the conjugated system. This disrupted π-π stacking between the fluorophores, thus leading to the phenomenon known as TIDA.

**Fig. 4:**
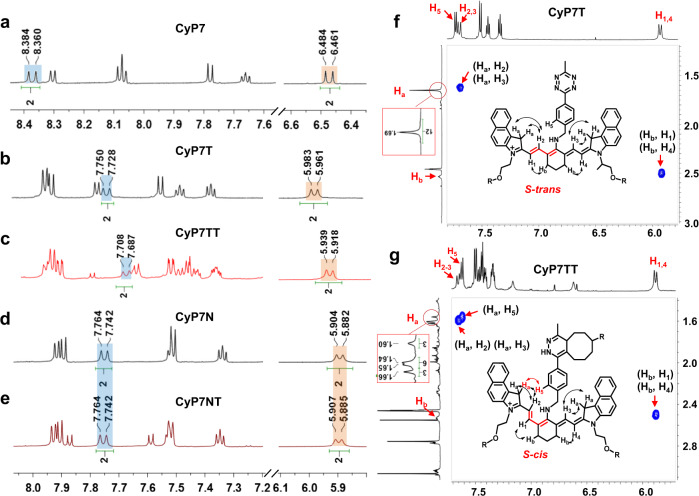
^1^H and 2 D ROESY NMR analysis. ^1^H NMR shift changes of vinyl-hydrogens (H_1_, H_2_, H_3_, H_4_) for **a** CyP7, **b** CyP7T, **c** CyP7TT, **d** CyP7N, and **e** CyP7NT. Orange regions refer to H_1_/H_4_ and blue regions refer to H_2_/H_3_. 2D ROESY NMR of **f** CyP7T and **g** CyP7TT in DMSO-*d*_*6*_.

Next, both 1D and 2D NMR analysis was carried out to study the structures and conformations of CyP7, CyP7T/CyP7TT, and CyP7N/CyP7NT. As shown in Fig. 4a, the H_2_/H_3_ and H_1_/H_4_ vinyl protons of CyP7 are located at 8.372 ppm and 6.473 ppm, respectively. As for CyP7T/CyP7TT and CyP7N/CyP7NT, the vinyl protons are all located further upfield (H_2_/H_3_ = 7.665-7.750; H_1_/H_4_ = 5.896-5.983), confirming a loss of planarity and resonance structure as previously shown in DFT calculations (Figs. 3 and 4b–e). In Fig. 4d–e, the vinyl proton peaks of Tz-TCO conjugate CyP7NT, remained relatively unchanged (H_2_/H_3_ = 7.764 ppm; H_1_/H_4_ = 7.742 ppm) after bioorthogonal ligation, corresponding to minimal perturbation in its conformation as evident by DFT calculations and fluorescence quenching measurements. However, for the Tz-TCO conjugate CyP7TT, the vinyl protons were shifted upfield (H_2_/H_3_ = 7.665; H_1_/H_4_ = 5.896 ppm) compared to CyP7T (H_2_/H_3_ = 7.750 ppm; H_1_/H_4_ = 5.983 ppm) (Fig. 4b,c). To further elucidate the 2D conformation of CyP7T and CyP7TT, rotating frame overhause effect spectroscopy (ROESY) NMR analysis was also conducted. As shown in Fig. 4f, Supplementary Figs. 29 and 30, the –CH_3_ protons of CyP7T (H_a_) exhibited strong nuclear overhauser effect with vinyl protons H_2_/H_3_, suggesting a *S-trans* conformation. Moreover, only singlet peaks were found for –CH_3_ protons (H_a_) belonging to CyP7T (as well as CyP7N and CyP7NT), clearly indicating a symmetrical *S-trans* conformation. In CyP7TT, however, the H_a_ CH_3_ proton showed strong correlation with the H_5_ aromatic proton on the Tz moiety, arising from an unsymmetrical *S-cis* conformer. ^1^H NMR analysis also revealed that the –CH_3_ protons (H_a_) in CyP7TT appeared as three separate peaks (Fig. 4g). Collectively, from these data, we can conclude that CyP7TT indeed adopted the uncommon *S-cis* conformer. Interestingly, DFT calculations similarly showed conformational transformation of the heptamethine chain from *S-trans* to *S-cis* occurred in CyP7TT after Tz-TCO ligation (Figs. 3a and 4f–g). Therefore, we concluded that *S*-trans-*S*-cis transformation with increased torsion angle reduced π-π stacking within the conjugated system after Tz-TCO ligation, then leading to TIDA.

To gain deeper insights into the TIDA effect on π-π stacking, molecular dynamic simulation was also performed. As shown in Supplementary Fig. 31, CyP7 formed face-to-face dimer-aggregates with strong attraction as indicated by the high binding energy (BE = –10.1558 kcal mol^–1^) and shortest coordination-shell distance (*r* = 0.496 nm). However, dimer interaction in CyP7TT was significantly impeded (BE = –1.8647 kcal mol^–1^, *r* = 0.518 nm) when compared with CyP7T (BE = –2.0121 kcal mol^–1^, *r* > 1 nm), further confirming disruption in π–π stacking. This molecular dynamic simulation data also concurred with the absorption/emission analysis result in Fig. 2d, whereby the disappearance of dimer peaks in absorption was observed (Fig. 2d). In addition, the spatial distribution functions (SDFs) of CyP7, CyP7T, and CyP7TT were also compared to analyze dimer formation (Supplementary Fig. 32). The three-dimensional density distribution on the conjugation plane of CyP7 was observed (Supplementary Fig. 32a, 1–4). This indicated the presence of strong π–π stacking which promotes dimer formation. Although CyP7T could form aggregates, π-π stacking of the cyanine backbone was seldom observed in the SDF (Supplementary Fig. 32b). As for CyP7TT, few regions of density distribution were observed, indicating aggregation or dimer formation was hardly observed (Supplementary Fig. 32c). In all, the theoretical calculations, molecular simulation, NMR characterization, and analysis provided a comprehensive understanding and deeper insight into the TIDA phenomenon. Therefore, the TIDA strategy presents a paradigm for designing bioorthogonally activatable NIR fluorophores for imaging applications.

### In vitro imaging

As proof-of-concept, bioorthogonally activatable imaging in live cells was next carried out. First, 4T1 cells were incubated with CyP7T, alongside amphiphilic CyP7, hydrophilic ICG, or hydrophobic IR780 to evaluate the membrane permeability of the probes. After 0.5 h incubation, fluorescence-activated cell sorting (FACS) analysis was used to determine cellular uptake of the different probes. For CyP7 and CyP7T, the cellular uptake was 99.98% and 100% respectively, indicating the excellent permeability and fast uptake of both dyes (Supplementary Fig. 33). CyP7T and CyP7TT also showed minimal cell cytotoxicity (Supplementary Fig. 34). Next, live-cell imaging was carried out on 4T1 cells treated with CyP7T, alongside CyP7 or ICG, using confocal laser scanning microscopy (CLSM). As shown in Supplementary Fig. 35, negligible fluorescence signal was observed for ICG after 0.5 h incubation. Obvious fluorescence signal was observed for CyP7, congruent with results from FACS analysis (Supplementary Fig. 33). For CyP7T-treated cells, stronger fluorescence signals were observed compared to CyP7-treated cells. This could be attributed to the larger Stokes shift of CyP7T, noting that TIDA could easily be realized for CyP7T because high autofluorescence can be fully minimized. For TCO/CyP7T-treated cells, CLSM analysis indicated strong fluorescence signal in the cytosol after 0.5 h incubation of CyP7T. The above results demonstrated that successful intracellular Tz-TCO ligation and TIDA resulted in significant fluorescence enhancement.

**Fig. 5:**
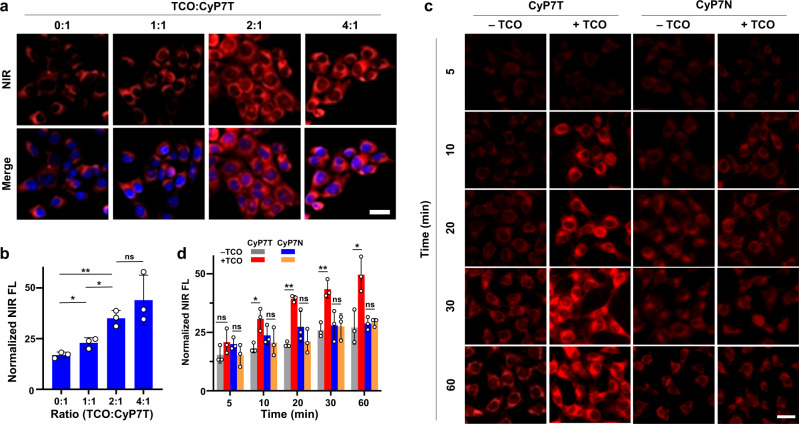
Bioorthogonal imaging of CyP7T in response to TCO in 4T1 cells. **a** CLSM imaging of 4T1 cells pre-incubated with different concentrations of TCO (0, 5, 10, 20 μM) for 3 h, washed with PBS, followed by incubation with CyP7T (5 μM) for 0.5 h. Cell nuclei were stained with DAPI (blue). **b** Qualitative analysis of fluorescence in Fig. 5a. Data are presented as mean values ± SD. A two-sided student’s t-test was performed (1:1 vs 0:1, *p* = 0.0244; 2:1 vs 1:1, *p* = 0.0102; 2:1 vs 0:1, *p* = 0.0015; 4:1 vs 2:1, *p* = 0.2988). **c** Confocal fluorescence images of 4T1 cells pre-incubated with 10 μM TCO for 3 h, washed with PBS, followed by incubation with 5 μM CyP7T or CyP7N for 5, 10, 15, 30 min. **d** Qualitative analysis of fluorescence in Fig. 5c. Data are presented as mean values ± SD. A two-sided student’s *t* test was performed (*p* = 0.2205, 0.1959, 0.0139, 0.5054, 0.0025, 0.2600, 0.0033, 0.9434, 0.0188, and 0.8194 from left to right). Fluorescence images of cells were recorded at *λ*_ex_ = 405 nm for DAPI, *λ*_ex_ = 640 nm for NIR dyes. Scale bar: 30 μm. Error bars: standard deviation from three separate measurements. *p < 0.05, **p < 0.01, ns not significant.

Once the proof-of-concept was successful, the optimal ratio of TCO:CyP7T for Tz-TCO ligation for use in subsequent live cells experiments was next determined by varying the ratio of TCO while keeping the concentration of CyP7T constant (5 μM). At 2:1 ratio of TCO:CyP7T, both CLSM imaging and qualitative analysis confirmed maximum fluorescence intensity was attained (Fig. 5a–b). We then evaluated the efficiency and effectiveness of TCO bioorthogonal activation in live cells incubated with CyP7T. 4T1 cells were first treated with 10 μM TCO for 3 h, followed by washing and incubating the cells with 5 μM CyP7T. CLSM images were then captured at different time points (5 to 30 min) post-CyP7T incubation. Notably, fluorescence enhancement could already be observed just 10 min after CyPT7 incubation (Fig. 5c). With longer incubation time up to 30 mins, CLSM images showed statistically significantly stronger fluorescence signals in cells treated with both TCO/CyP7T (Fig. 5d). As for negative control cells, the fluorescence signal showed no significant difference upon bioorthogonal ligation (Fig. 5c, d, Supplementary Figs. 36 and 37).

**Fig. 6:**
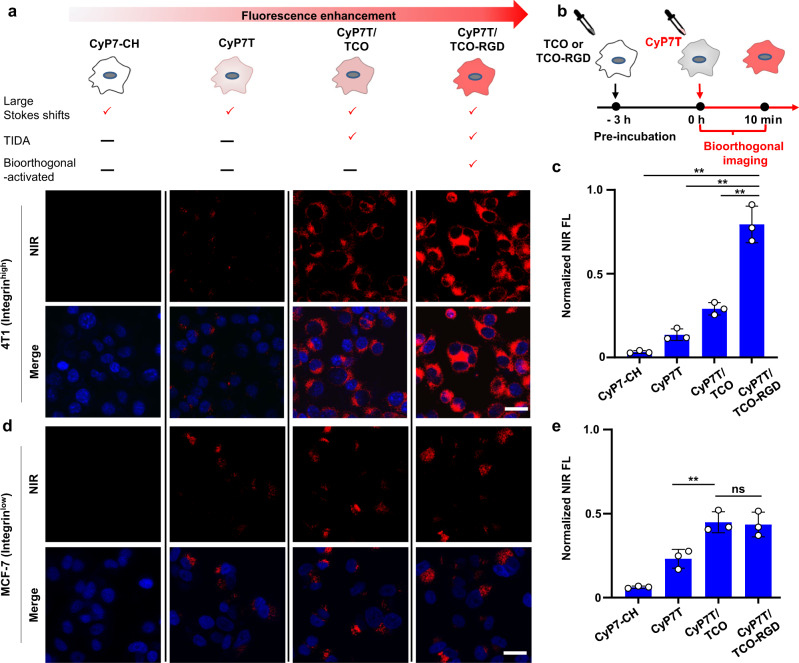
Bioorthogonal-activated imaging in 4T1 cells and MCF-7 cells. **a** CLSM imaging of 4T1 cells (integrin *α*_*v*_*β*_*3*_ high-expressing) incubated with CyP7T, CyP7T/TCO, and CyP7T/TCO-RGD (5 μM) for 0.5 h. **b** Timeline for the addition of CyP7T and TCO/TCO-RGD. Cells were pre-incubated with 10 μM TCO or TCO-RGD for 3 h, washed with PBS, followed by incubation with CyP7T (5 μM) for 10 min. **c** Quantification of NIRF signal in **a**. Data are presented as mean values ± SD. A two-sided student’s *t* test was performed (CyP7T/TCO-RGD vs CyP7-CH, *p* = 0.0003; CyP7T/TCO-RGD vs CyP7T, *p* = 0.0006; CyP7T/TCO-RGD vs CyP7T/RGD, *p* = 0.0017). **d** CLSM imaging of MCF-7 cells (integrin *α*_*v*_*β*_*3*_ low-expressing) incubated with CyP7T, CyP7T/TCO, and CyP7T/TCO-RGD (5 μM) for 0.5 h. **e** Quantification of NIRF signal in **d**. Data are presented as mean values ± SD. A two-sided student’s *t* test was performed (CyP7T/TCO vs CyP7T, *p* = 0.0187; CyP7T/TCO vs CyP7T/TCO-RGD, *p* = 0.8359). Cells were stained with nuclear dye, DAPI (blue). All the images were acquired at 40× magnification. Scale bar: 30 μm. Error bars: standard deviation from three separate measurements. **p < 0.01, ns not significant.

To further evaluate the bioorthogonally activatable enhancement feature of CyP7T, targeted imaging was carried out on 4T1 cells (Fig. 6a). 4T1 breast cancer cells are known to high-express integrin *α*_*v*_*β*_*3*_ receptors. The tri-amino acid sequence, arginine-glycine-aspartate or “RGD” is commonly used as an inhibitor of integrin *α*_*v*_*β*_*3*_ receptor-ligand interactions to induce apoptosis. RGD peptide was conjugated to TCO, endowing it with tumor-targeting property^43^. After that, 4T1 cells were first pre-incubated with 10 μM TCO-RGD for 3 h, followed by washing to remove excess TCO-RGD, and subsequent incubation with 5 μM CyP7T (Fig. 6b). For TCO-RGD-treated cells, strong fluorescence signal could be observed by CLSM analysis only after 10 min incubation of CyP7T, demonstrating that bioorthogonally activatable imaging resulted in significant fluorescence enhancement. Qualitative analysis of fluorescence in Fig. 6a indicated that CyP7T showed a ca. 6-fold increase in fluorescence intensity in the presence of TCO-RGD (Fig. 6c). Meanwhile, the negative cell experiments were carried out on the *α*_*v*_*β*_*3*_-low expressing MCF-7 cells^62^, TCO-RGD-treated cells showed no significantly fluorescence difference compared to TCO-treated cells by CLSM analysis (Fig. 6d–e). Therefore, these results confirmed successful and efficient bioorthogonal-activated NIR imaging with enhanced fluorescence signals using CyP7T in live cells.

### In situ and in vivo imaging

With success in live cells experiments, the use of CyP7T was next applied in in vivo imaging. First, the biodistribution and tumor accumulation of PEG-modified CyP7 was evaluated alongside hydrophobic CyB7 and hydrophilic ICG in 4T1 tumor-bearing mice. As shown in Supplementary Figs. 38 and 39, ICG-treated mice group showed poor tumor accumulation with elimination half-life, t_1/2_ = 1.65 min. On the other hand, PEG-modified CyP7 showed both good tumor accumulation and longer half-life, t_1/2_ = 24.9 min. After 24 h post-injection (HPI), ex vivo fluorescence images of major organs were also obtained to examine the biodistribution of probes (Supplementary Fig. 40). PEG-modification of CyP7 endowed the probe with good in vivo properties essential for reducing background signals during imaging and eliminating toxicity due to accumulation in the liver.

**Fig. 7:**
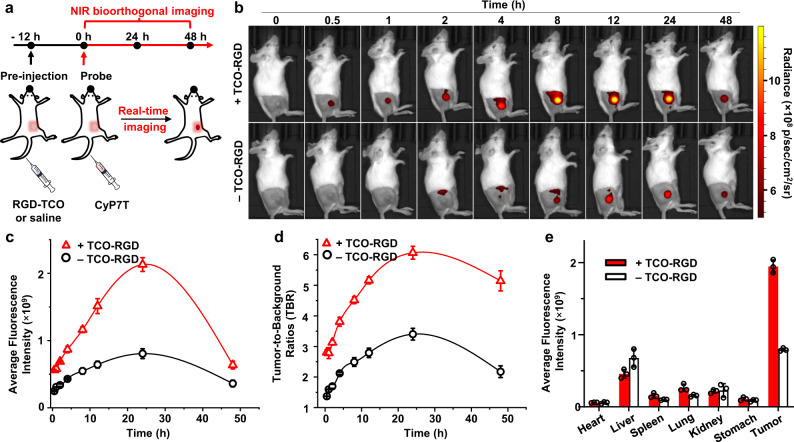
In vivo bioorthogonal NIR imaging for 4T1 tumor-bearing mice. **a** Timeline of RGD-TCO, CyP7T injection, and NIR imaging. Mice were pre-injected with TCO-RGD (10 nmol) or saline, followed by injection of CyP7T (5 nmol) 12 h later. **b** NIR imaging was performed at various timepoints (0, 0.5, 1, 2, 4, 8, 12, 24, 48 h) post-injection of CyP7T. **c** Fluorescence signal intensity in tumor tissue and **d** Tumor-to-background ratio (TBR) over time, *n* = 3. TBR was determined by (tumor ROI—background ROI)/background ROI. **e** Fluorescence intensity analysis of excised organs harvested from mice 24 h post-injection of CyP7T. Data are presented as mean ± SD. (*n* = 3 biologically independent mice per group).

Bioorthogonally activatable imaging using CyP7T was next validated in 4T1 tumor-bearing mouse model. First, to validate the fluorescence enhancement in vivo, intratumor administration was chosen to eliminate differences in the properties of the probes as a result of metabolism or clearance arising from the circulatory system. TCO-RGD and CyP7T or CyP7N probes were simultaneously administered into 4T1 tumor-bearing mice intratumorally. The region-of-interest (ROI) was employed to quantitate fluorescence signals in the tumor. After 15 min intratumoral injection, fluorescent signals in tumors of CyP7T/TCO-RGD groups showed twofold fluorescent enhancement compared to CyP7T only group until 24 HPI (Supplementary Fig. 41). As for the negative control CyP7N and CyP7N/TCO-RGD groups, no statistically significant difference in fluorescence signals was observed. Next, intravenous (i.v.) administration of TCO-RGD and the cyanine probes was carried out. After 12 h post i.v. injection of TCO-RGD, CyP7T was subsequently administered and in vivo imaging was performed simultaneously (Fig. 7a). As shown in Supplementary Fig. 42, fluorescence signals could be clearly observed at the tumor site as early as 5 min. However, mice in the control group injected with only CyP7T achieved the same fluorescence intensity at the tumor site only after 8 HPI (Fig. 7b, c). ROI analysis showed that fluorescence signals in tumors of both TCO-RGD-treated mice and control groups gradually increased, reaching a maximum after 24 HPI. The ROI signal in tumor region of TCO-RGD pre-treated mice was 2.6-fold higher than that for the control group mice (Fig. 7c). In the presence of TCO-RGD, the tumor-to-background ratio (TBR) of fluorescence signals was about 6 and 4 after 24 HPI and 48 HPI, respectively (Fig. 7d). In contrast, the TBR in control group mice was approximately 3 at 24 HPI and decreased to 2 at 48 HPI. Excised organs were harvested for ex vivo fluorescence imaging and biodistribution studies after 24 HPI (Supplementary Fig. 43). Semi-quantitative analysis demonstrated that tumors showed the highest fluorescence signal, followed by livers for both groups (Fig. 7e). Notably, TCO-RGD-treated group showed higher tumor-to-liver fluorescence signal ratio (4.6-fold) compared to control group mice (1.2-fold). Next, the biocompatibility and biosafety of the probe were evaluated by histology. Hematoxylin and eosin stain (H&E)-stained images of major organ sections displayed no obvious pathological changes or structural abnormalities in the heart, liver, spleen, kidney, lung, and stomach (Supplementary Fig. 44). Together, these results confirmed that the bioorthogonally activatable cyanine probe CyP7T, can be safely and successfully used to potentiate sensitivity and specificity of NIR imaging both in live cells as well as in vivo.

## Discussion

In summary, we have developed a bioorthogonally activatable NIR cyanine probe (CyP7T) using TIDA for in vivo tumor imaging. DFT calculations, molecular simulations, and NMR analysis confirmed that upon Tz-TCO ligation, conformational change on the heptamethine chain of the fluorophore from *S-trans* to *S-cis* resulted in TIDA, leading to significant fluorescence enhancement. We demonstrated that sensitive NIR fluorescence imaging could be carried out in live cells, with visualization as fast as within 5 min. Similarly in vivo, the fluorescence of CyP7T could be successfully and efficiently activated by bioorthogonal reaction with TCO-RGD at tumor sites, within 5 min. The sensitivity and specificity for the tumor detection are highly dependent on the tumor-to-background ratio. It is well-documented that the optimal threshold value for tumor-to-background ratio was ≥3.0^6^. For 700‒900 nm NIR range, the TBR ratios were typically ~2‒4 by using organic fluorophores^63,64^. In our work, on 4T1 tumor-bearing mice model, bioorthogonal imaging using CyP7T and TCO-RGD afforded high tumor-to-background ratio of 6 for sensitive and specific in vivo tumor imaging. To the best of our knowledge, the TIDA strategy is different from other known designs based on Tz-quenching mechanisms such as FRET, TBET, PET, and ICT. Thus, this study presents a paradigm in the design of bioorthogonally activatable NIR fluorescence probes. We foresee that such a strategy would pave the way for development of other fluorescence activatable scaffolds for bioorthogonal chemistry and help advance bioorthogonal chemistry in vivo imaging and diagnosis.

## Methods

### General measurements

TCO-RGD was synthesized according to our previous study^43^. High-resolution mass spectra (HRMS) were performed on a Bruker OTOF-Q II mass spectrometer. ^1^H NMR spectra were recorded with a Varian 600 MHz NMR with trimethylchlorosilane as an internal standard. The fluorescence spectra were measured in an Edinburgh Instruments’ FLS1000. The imaging experiments in vitro were recorded on an Olympus FV 10i confocal fluorescent microscope, and the fluorescent signals of DAPI were recorded by blue channel (excitation 405 nm, emission 461 nm) and NIR dyes by red channel (excitation 640 nm, emission 665 nm). In vivo fluorescence imaging analysis was carried out in an IVIS Kinetic imaging system (Living Image Software version 4.5, excitation 660 nm, emission 710 nm). FACS analysis was detected with a BD Accuri C6 flow cytometry (BD CFlow Plus Software version 1.0.264.21), and fluorescent signals were recorded by FL4 channel (excitation 640 nm, emission 675 nm).

### Synthesis of CyP7

A mixture of 1,1,2-trimethylbenz[e]indole (0.6 g, 2.9 mmol, 1 eq.) and diethylene glycol 2-bromoethyl methyl ether (2.6 g, 11.6 mmol, 4 eq.) in acetonitrile (8 mL) was heated in an oil bath at 80 °C for 48 h. After cooling to room temperature, the solvent was removed by rotary evaporation. The crude compound was washed with ether (5 mL ×3) and dried to afford the quaternary ammonium salt as a dark purple solid (yield: 60%). Quaternary ammonium salt (0.5 g, 1.1 mmol, 2.1 eq), 2-chloro-1-formyl-3-hydroxymethylenecyclohexene (0.09 g, 0.55 mmol, 1 eq.) and sodium acetate (0.09 mg, 1.1 mmol, 2 eq.) were dissolved in 8 mL acetic anhydride, then heated at 80 °C for 3 h. The progress of the reaction was monitored by TLC. After completion of reaction, the mixture was poured into ice water for quenching. CH_2_Cl_2_ was added to extract the crude product. The organic extracts were combined and then concentrated to dryness. The resulting solid product was purified by flash column chromatography (methyl alcohol/dichloromethane, 1/50, v/v). The purified product, CyP7, was obtained as green semisolid (0.3 g, 60%). ^1^H NMR (600 MHz, DMSO-*d*_*6*_): 1.87–1.91 (m, 2H, CH_2_-CH_2_-CH_2_), 1.96 (s, 12H, CH_3_CCH_3_), 2.74 (t, 4H, *J* = 6.0 Hz, CH_2_-CH_2_-CH_2_), 3.09 (s, 6H, CH_3_), 3.20 (t, 4H, *J* = 6.0 Hz, CH_2_-), 3.32 (t, 4H, *J* = 6.0 Hz, CH_2_-), 3.39–3.40 (m, 4H, CH_2_-), 3.52 (t, 4H, *J* = 6.0 Hz, CH_2_-), 3.86 (t, 4H, *J* = 6.0 Hz, CH_2_-), 4.57 (t, 4H, *J* = 6.0 Hz, NCH_2_-), 6.47 (d, 2H, *J* = 14.4 Hz, CH = CH), 7.53 (t, 2H, *J* = 8.0 Hz, ArH), 7.75 (t, 2H, *J* = 8.0 Hz, ArH), 7.78 (d, 2H, *J* = 8.0 Hz, ArH), 8.07 (t, 4H, *J* = 8.0 Hz, ArH), 8.30 (d, 2H, *J* = 8.0 Hz, ArH), 8.37 (d, 2H, *J* = 14.4 Hz, CH = CH). ^13^C NMR (150 MHz, DMSO-*d*_*6*_) δ (ppm): 173.66, 146.60, 141.31, 139.62, 132.82, 130.95, 129.65, 129.39, 127.22, 126.87, 125.76, 124.46, 121.76, 111.73, 101.62, 70.62, 69.81, 69.29, 69.05, 67.25, 57.41, 50.12, 44.01, 26.54, 25.44, 19.95. High-resolution MS (ESI, positive ion mode) *m*/*z*: calculated *M*_*r*_ = 847.4453 for C_52_H_64_ClN_2_O_6_^+^, found *m*/*z* = 847.4448 ([*M*_*r*_]).

### Synthesis of CyP7T

CyP7 (50 mg, 53 μmol, 1 eq.) and methytetrazine-amine HCl salt (63 mg, 265 μmol, 5 eq.) were dissolved in 3 mL *N,N*-dimethylformamide. Then, triethylamine (25 μL) was added dropwise to the reaction mixture and further stirred at 80 °C for 2 h. The progress of the reaction was monitored by TLC. After completion of reaction, the mixture was poured into ice water for quenching. CH_2_Cl_2_ was added to extract the crude product. The organic extracts were combined and concentrated to dryness. The resulting solid product was purified by flash column chromatography (methyl alcohol/dichloromethane, 1/50, v/v). The purified product, CyP7T, was obtained as blue semisolid (41 mg, 72%). ^1^H NMR (600 MHz, DMSO-*d*_*6*_): 1.70 (s, 12H, CH_3_CCH_3_), 1.79‒1.83 (quint, 2H, *J* = 6.0 Hz, CH_2_-CH_2_-CH_2_), 2.58 (t, 4H, *J* = 6.0 Hz, CH_2_-CH_2_-CH_2_), 3.01 (s, 3H, CH_3_), 3.10 (s, 6H, CH_3_), 3.22 (t, 4H, *J* = 6.0 Hz, CH_2_-), 3.35‒3.36 (m, 4H, CH_2_-), 3.41 (t, 4H, *J* = 6.0 Hz, CH_2_-), 3.51 (t, 4H, *J* = 6.0 Hz, CH_2_-), 3.79 (t, 4H, *J* = 6.0 Hz, CH_2_-), 4.29 (t, 4H, *J* = 6.0 Hz, CH_2_-), 5.03 (d, 2H, *J* = 6.0 Hz, CH_2_-), 5.76 (s, 1H, NH), 5.97 (d, 2H, *J* = 13.2 Hz, CH = CH), 7.38 (t, 2H, *J* = 8.0 Hz, ArH), 7.49 (t, 2H, *J* = 8.0 Hz, ArH), 7.55 (d, 2H, *J* = 8.4 Hz, ArH), 7.74 (d, 2H, *J* = 13.2 Hz, CH = CH), 7.77 (d, 2H, *J* = 8.4 Hz, ArH), 7.92–7.96 (dd, 6H, *J*_*1*_ = 9.0 Hz, *J*_*2*_ = 9.0 Hz, ArH), 8.60 (d, 2H, *J* = 8.4 Hz, ArH). HRMS: calculated *M*_*r*_ = 1012.5701 for C_62_H_74_N_7_O_6_^+^, found *m*/*z* = 1012.5607 ([*M*_*r*_]).

### Synthesis of CyP7N

CyP7N was synthesized using CyP7 (50 mg, 53 μmol, 1 eq.) and methyltetrazine-PEG4-Amine (96 mg, 265 μmol, 5 eq.) by the similar method of CyP7T. The purified product, CyP7N, was obtained as blue semisolid (44 mg, 67%). ^1^H NMR (600 MHz, DMSO-*d*_*6*_): 1.72 (t, 2H, *J* = 6.0 Hz, CH_2_-CH_2_-CH_2_-), 1.86 (s, 12H, CH_3_CCH_3_), 1.92‒1.98 (m, 4H, CH_2_-CH_2_-CH_2_-), 2.91 (s, 3H, CH_3_), 3.06 (s, 6H, CH_3_), 3.12-3.13 (m, 4H, CH_2_-), 3.19 (t, 4H, *J* = 4.8 Hz, CH_2_-), 3.31 (t, 4H, *J* = 4.8 Hz, CH_2_-), 3.36‒3.38 (m, 4H, CH_2_-), 3.48 (t, 4H, *J* = 4.8 Hz, CH_2_-), 3.56–3.59 (m, 4H, CH_2_-), 3.62‒3.64 (t, 2H, *J* = 4.8 Hz, CH_2_-), 3.71 (t, 2H, *J* = 4.8 Hz, CH_2_-), 3.75 (t, 4H, *J* = 4.8 Hz, CH_2_-), 3.83 (t, 2H, *J* = 4.8 Hz, CH_2_-), 4.11 (t, 2H, *J* = 4.8 Hz, CH_2_-), 4.25 (t, 4H, *J* = 4.8 Hz, CH_2_-), 5.89 (d, 2H, *J* = 13.2 Hz, CH = CH), 7.06 (d, 2H, *J* = 9.0 Hz, ArH), 7.34 (t, 2H, *J* = 8.4 Hz, ArH), 7.52 (t, 4H, *J* = 9.0 Hz, ArH), 7.75 (d, 2H, *J* = 13.2 Hz, CH = CH), 7.89‒7.93 (dd, 4H, *J*_*1*_ = 8.4 Hz, *J*_*2*_ = 9.0 Hz, ArH), 8.09 (d, 2H, *J* = 8.4 Hz, ArH), 8.30 (d, 2H, *J* = 9.0 Hz, ArH). ^13^C NMR (150 MHz, DMSO-*d*_*6*_) δ (ppm): 174.81, 169.75, 167.07, 163.43, 162.43, 141.52, 138.62, 131.09, 130.93, 130.32, 130.17, 130.08, 129.67, 128.31, 127.76, 124.56, 124.07, 122.20, 120.68, 115.79, 111.91, 95.42, 71.69, 70.87, 70.32, 70.12, 69.34, 67.95, 58.48, 49.13, 46.23, 35.64, 31.82, 30.30, 28.22, 27.08, 25.64, 25.21, 22.63, 21.91, 21.25. HRMS: calculated *M*_*r*_ = 1174.6587 for C_69_H_88_N_7_O_10_^+^, found *m*/*z* = 1174.6521 ([*M*_*r*_]).

### Synthesis of CyP7TT

CyP7T (10 mg, 9 μmol, 1 eq.) and TCO-NHS (5 mg, 18 μmol, 2 eq.) (abbreviated as TCO) were dissolved in 3 mL DMSO and the mixture was further stirred at room temperature for 0.5 h. After completion of the reaction, the mixture was washed with ether (5 mL ×3) to obtain the crude product. The solid was lyophilized using a freeze-dryer (Christ Alpha 1-2 LDplus) to yield CyP7TT as blue semisolid (11 mg, 95%). ^1^H NMR (600 MHz, DMSO-*d*_*6*_): 1.60‒1.66 (m, 12H, CH_3_CCH_3_), 2.09‒2.13 (m, 2H, CH_2_-CH_2_-CH_2_-), 2.57 (t, 4H, *J* = 6.0 Hz, CH_2_-), 2.59 (s, 3H, CH_3_), 2.81 (t, 4H, *J* = 6.0 Hz, CH_2_-), 3.06‒3.09 (m, 4H, CH_2_-), 3.11 (s, 6H, CH_3_), 3.23 (t, 4H, *J* = 6.0 Hz, CH_2_-), 3.35 (t, 4H, *J* = 6.0 Hz, CH_2_-), 3.41 (t, 4H, *J* = 6.0 Hz, CH_2_-), 3.50‒3.52 (m, 4H, CH_2_-), 3.77‒3.79 (m, 4H, CH_2_-), 4.03 (t, 1H, *J* = 6.0 Hz, CH), 4.27 (t, 4H, *J* = 6.0 Hz, CH_2_-), 4.41‒4.44 (dd, 4H, *J* = 6.0 Hz, CH_2_-), 4.91‒4.95 (m, 2H, CH_2_), 5.31‒5.33 (m, 1H, CH), 5.46‒5.50 (m, 2H, CH_2_-), 5.60‒5.65 (m, 2H, CH_2_-), 5.93(d, 2H, *J* = 12.6 Hz, CH=CH), 7.37–7.40 (m, 2H, ArH), 7.44‒7.46 (m, 1H, ArH), 7.48 (d, 2H, *J* = 8.4 Hz, ArH), 7.51 (d, 1H, *J* = 8.4 Hz, ArH), 7.54 (d, 2H, *J* = 9.0 Hz, ArH), 7.66 (d, 2H, *J* = 8.4 Hz, ArH), 7.69 (d, 2H, *J* = 12.6 Hz, CH = CH), 7.92 (d, 2H, *J* = 9.0 Hz, ArH), 7.95 (d, 4H, *J* = 8.4 Hz, ArH), 9.46 (s, 1H, NH), 10.53 (s, 1H, NH). ^13^C NMR (150 MHz, DMSO-*d*_*6*_) δ (ppm): 173.32, 170.45, 169.71, 160.91, 158.20, 151.05, 142.64, 141.37, 138.28, 137.71, 137.26, 131.24, 130.88, 130.27, 130.17, 130.01, 128.31, 128.09, 127.51, 124.12, 122.60, 122.03, 111.93, 111.81, 95.46, 71.69, 70.88, 70.32, 70.13, 67.95, 58.48, 49.32, 43.71, 31.81, 30.91, 29.85, 29.53, 29.23, 29.10, 27.79, 27.70, 27.61, 23.08, 22.62, 21.81, 20.66, 18.91. HRMS: calculated *M*_*r*_ = 1251.6740 for C_75_H_91_N_6_O_11_^+^, found *m*/*z* = 1251.6746 ([*M*_*r*_]).

### Synthesis of CyP7NT

CyP7N (11 mg, 9 μmol, 1 eq.) and TCO-NHS (5 mg, 18 μmol, 2 eq.) were dissolved in 3 mL DMSO and the mixture was further stirred at room temperature for 0.5 h. After completion of the reaction, the mixture was washed with ether (5 mL ×3) to obtain the crude product. The solid was lyophilized using a freeze-dryer to give CyP7NT as a blue semisolid (13 mg, 96%). ^1^H NMR (600 MHz, DMSO-*d*_*6*_): 1.86 (s, 12H, CH_3_CCH_3_), 1.93‒2.04 (m, 6H, CH_2_-), 2.04‒2.10 (m, 2H, CH_2_-CH_2_-CH_2_-), 2.22‒2.25 (m, 2H, CH_2_-), 2.27‒2.32 (m, 4H, CH_2_-), 2.48 (t, 4H, *J* = 6.0 Hz, CH_2_-CH_2_-CH_2_-), 2.55 (s, 3H, CH_3_), 3.07 (s, 6H, CH_3_), 3.19 (t, 4H, *J* = 4.8 Hz, CH_2_-), 3.31‒3.32 (m, 4H, CH_2_-), 3.36‒3.38 (m, 4H, CH_2_-), 3.47‒3.48 (m, 4H, CH_2_-), 3.52‒3.54 (m, 4H, CH_2_-), 3.57 (t, 2H, *J* = 4.8 Hz, CH_2_-), 3.62 (t, 2H, *J* = 4.8 Hz, CH_2_-), 3.65‒3.68 (m, 2H, CH_2_-), 3.75 (t, 4H, *J* = 4.8 Hz, CH_2_-), 3.83 (t, 2H, *J* = 4.8 Hz, CH_2_-), 3.97‒3.99 (m, 1H, CH), 4.03‒4.07 (m, 1H, CH), 4.25 (s, 4H, CH_2_-CH_2_-CH_2_-), 4.37-4.39 (m, 2H, CH_2_-), 5.41‒5.46 (m, 2H, CH_2_-), 5.55‒5.60 (m, 2H, CH_2_-), 5.90 (d, 2H, *J* = 13.2 Hz, CH=CH), 6.79 (d, 1H, *J* = 9.0 Hz, ArH), 6.88 (d, 1H, *J* = 9.0 Hz, ArH), 7.35 (t, 2H, *J* = 7.8 Hz, ArH), 7.52 (d, 3H, *J* = 9.0 Hz, ArH), 7.59 (d, 1H, *J* = 9.0 Hz, ArH), 7.75 (d, 2H, *J* = 13.2 Hz, CH = CH), 7.87 (d, 1H, *J* = 9.0 Hz, ArH), 7.94–7.90 (dd, 4H, *J*_*1*_ = 8.4 Hz, *J*_*2*_ = 7.8 Hz, ArH), 8.09 (d, 3H, *J* = 9.0 Hz, ArH), 9.08 (s, 1H, NH), 10.50 (s, 1H, NH). HRMS: calculated *M*_*r*_ = 1413.7632 for C_82_H_105_N_6_O_15_^+^, found *m*/*z* = 1413.7634 ([*M*_*r*_]).

### Synthesis of CyP7-CH

CyP7-CH was synthesized using CyP7 (50 mg, 53 μmol, 1 eq.) and cyclohexylamine (30 μL, 265 μmol, 5 eq.), similar to the synthesis of CyP7T. The purified product, CyP7-CH, was obtained as a blue semisolid (25 mg, 51%). ^1^H NMR (600 MHz, DMSO-*d*_*6*_): 1.26–1.28 (m, 4H, CH_2_-), 1.75–1.78 (m, 4H, CH_2_-), 1.93 (s, 12H, CH_3_CCH_3_), 2.53 (t, 4H, *J* = 6.0 Hz, CH_2_-), 3.12 (s, 6H, CH_3_), 3.25 (t, 4H, *J* = 4.8 Hz, CH_2_-), 3.36–3.38 (m, 11H, CH_2_-, CH-), 3.43 (t, 4H, *J* = 4.8 Hz, CH_2_-), 3.54 (t, 4H, *J* = 4.8 Hz, CH_2_-), 3.82 (t, 4H, *J* = 4.8 Hz, CH_2_-), 4.33 (s, 4H, CH_2_-), 6.00 (d, 2H, *J* = 13.2 Hz, CH=CH), 7.41 (t, 2H, *J* = 8.0 Hz, ArH), 7.57 (d, 1H, *J* = 8.0 Hz, ArH), 7.59 (d, 3H, *J* = 8.0 Hz, ArH), 7.82 (d, 2H, *J* = 13.2 Hz, ArH), 7.97 (t, 4H, *J* = 8.4 Hz, ArH), 8.21 (d, 2H, *J* = 8.4 Hz, CH=CH). ^13^C NMR (150 MHz, DMSO-*d*_*6*_) δ (ppm): 169.01, 167.49, 140.41, 137.89, 130.29, 129.92, 129.32, 129.06, 127.27, 126.74, 123.12, 121.42, 120.88, 110.92, 94.78, 70.65, 69.29, 69.24, 69.10, 66.97, 58.78, 57.44, 48.71, 48.52, 42.79, 33.61, 29.76, 27.08, 24.52, 24.31, 23.95, 21.09. HRMS: calculated *M*_*r*_ = 910.5729 for C_58_H_76_N_3_O_6_^+^, found *m*/*z* = 910.5736 ([*M*_*r*_]).

### In Vitro Fluorescence imaging

Approximately 1 × 10^5^ cells were seeded in a confocal imaging chamber and incubated overnight at 37 °C in 5% CO_2_. The following day, CyP7T/TCO and CyP7N/TCO samples were first pre-incubated with 10 μM TCO-NHS (in RPMI-1640 medium) for 3 h. After which, the medium was removed, and the cells were washed thrice with PBS buffer to remove excess TCO-NHS. Next, the cells were incubated with fresh RPMI-1640 medium containing CyP7T or CyP7N (5 μM) for 5, 10, 20, and 30 min. As for the other control samples, cells were incubated with 5 μM ICG, CyP7, CyP7-CH, CyP7T, CyP7N, and CyP7N_3_ for 0.5 h. Prior to CLSM imaging, the cells were washed with PBS buffer thrice, fixed with 4% paraformaldehyde, and nuclei staining was carried out using DAPI (10 μg/mL). Finally, fluorescence images of cells were recorded at *λ*_ex_ = 405 nm for DAPI and *λ*_ex_ = 675 nm for NIR dyes using a confocal laser scanning microscope.

### Flow cytometry analysis

4T1 cells were seeded on 24-well plates at the density of 1 × 10^5^ cells per well, and cultured for 12 h to grow adherently. The cells were incubated with ICG, CyB7, CyP7, and CyP7T at a concentration of 5 μM for 0.5, 1, 3, and 5 h, respectively. After culturing different times, then washed with PBS three times, the cells were digested, harvested, and quantitatively determined by flow cytometry.

### In vivo fluorescence imaging

All animal operations in this article are in compliance with relevant regulations and ethical requirements. Mice were maintained in standard housing conditions (10 h light/14 h dark cycle, 21−25 °C, 40−70% humidity). To develop the breast tumor model, 6–8-week SPF female healthy BALB/c mice were chosen, and 2 × 10^6^ murine breast cancer 4T1 cells were subcutaneously implanted into the right lower limb per mouse. Animals received care in accordance with the Guidance Suggestions for the Care and Use of Laboratory Animals Center of Fourth Military Medical University (20190226). All probes were dissolved in saline for injection. When the diameter of the tumor developed to be 4−6 mm after one-week inoculation, the mice were randomly divided into two groups (*n* = 3). The mice are injected with ICG, CyP7, or CyP7T (5 nmol) via tail vein. The bioorthogonally activatable group was injected with 10 nmol of TCO-RGD 12 h in advance, and then injected with 5 nmol of CyP7T. Mice were anesthetized with 5% isoflurane and then transferred to the IVIS imaging system (PerkinElmer IVIS Lumina III) to obtain fluorescence imaging at 1, 2, 4, 8, 12, 24, and 48 h after dye injection. ICG and CyP7 groups used a 780 nm excitation wavelength and 840 nm filter. CyP7T and CyP7N groups used a 680 nm excitation wavelength and 790 nm filter. Quantification of tumor fluorescence images was analyzed using the IVIS software.

### Blood circulation and clearance

Blood samples from BALB/c mice after i.v. injection of 50 nmol dye were taken at different time points (0, 2.5, 5, 15, 30 min, 1, 2, 3, 6 h). All the blood samples were stored in an anticoagulation tube (BD Vacutainer, K2EDTA), and then the fluorescent intensity of ICG (ex 790 nm/em 804 nm) and CyP7 (ex 760 nm/em 825 nm) was determined by Edinburgh Instruments’ FLS1000.

### Anatomical imaging and H&E experiments

After 24 h of continuous imaging of each group of mice, the mice were sacrificed and dissected by cervical dislocation method, and the fluorescent signals of major organs (heart, liver, spleen, lung, kidney, and stomach) and tumors were collected. For hematoxylin/eosin (H&E) experiments, the mice were sacrificed by cervical dislocation to dissect the main organs, which were placed in 4% paraformaldehyde solution for tissue fixation and then stained with H&E staining process.

### Data analysis

Results were presented as ±standard deviation (SD) from three separate measurements. Statistical significance was evaluated by two-tailed Student’s *t* test. The levels of significance were set at *p < 0.05, **p < 0.01, ns: not significant. All statistical analyses were performed by GraphPad Prism (version 8.0.2). Quantification analysis of NIRF signal in CLSM images was performed by ImageJ software (version 1.52a). Theoretical calculation and molecular dynamic simulation methods are provided in the Supplementary Information.

### Reporting summary

Further information on research design is available in the Nature Research Reporting Summary linked to this article.

## Supplementary information

Supplementary Information

Reporting Summary

## Data Availability

The spectrum, fluorescence loss properties, qualitative fluorescence in cell imaging, optimized Cartesian coordinates, and plane equations of the dyes are provided in the Source Data file. Most data supporting the findings in this study are available in the paper and Supplementary information files. The data that support the findings of this study are available from the corresponding author upon request. Source data are provided with this paper.

## Source data

Source Data
