# Supplementary material for: Bioorthogonally activatable cyanine dye with torsion-induced disaggregation for in vivo tumor imaging
Source: Nat Commun. 2022 Jun 18;13:3513. doi: 10.1038/s41467-022-31136-3 (PMC9206667; doi:10.1038/s41467-022-31136-3)
Supplement: Supplementary file 1 — Supplementary Information [file 41467_2022_31136_MOESM1_ESM.pdf]

Supplementary Information

Bioorthogonally Activatable Cyanine Dye with Torsion-Induced Disaggregation for In Vivo Tumor Imaging

Zhang et al.

Supplementary Methods

Materials. Methyltetrazine-Amine, Methyltetrazine-PEG4-Amine, (E)-Cyclooct-4-enyl 2,5-dioxo-1-pyrrolidinyl carbonate and TCO-carbonate Trans-Cyclooctene-NHS ester (TCO-NHS), 3-azido-1-Propanamine, endo-BCN-NHS ester (BCN-NHS) were purchased from Click Chemistry Tools. Diethylene glycol 2-bromoethyl methyl ether was purchased from Sigma-Aldrich. Other common reagents and solvents were purchased from Sigma-Aldrich, TCI Shanghai or J&K Chemical. PBS and DMEM were purchased from Thermo Fisher Scientific. Cell Counting Kit-8 (CCK-8) assay was purchased from Dojindo Molecular Technologies. Female BALB/C mice (4 weeks) were supplied by the BEIJING HFK BIOSCIENCE (Beijing, China). All animal experiments were performed in compliance with the Institutional Animal Care and Use Committee.

Synthesis of CyP7N$_3$. CyP7N$_3$ was synthesized using CyP7 (50 mg, 53 *μ*mol, 1 eq.) and 3-azido-1-propanamine (49 uL, 530 *μ*mol, 10 eq.), similar to the synthesis of CyP7T. The purified product, CyP7N$_3$, was obtained as a blue solid (20 mg, 40%). ^{1}H NMR (600 MHz, DMSO-d_6): 1.73 (t, 2H, *J* =6.0 Hz, CH$_2$-), 1.87 (s, 12H, CH$_3$CCH), 1.99-2.04 (m, 2H, CH$_2$-), 2.50 (t, 4H, *J* =6.0 Hz, CH$_2$-), 3.07 (s, 6H, CH$_3$), 3.20 (t, 4H, *J* =4.8 Hz, CH$_2$-), 3.32 (t, 4H, *J* =4.8 Hz, CH$_2$-), 3.38 (t, 4H, *J* =4.8 Hz, CH$_2$-), 3.48 (t, 4H, *J* =4.8 Hz, CH$_2$-), 3.53 (t, 2H, *J* =6.0 Hz, CH$_2$-), 3.75-3.80 (m, 6H, CH$_2$-), 4.26 (s, 4H, CH$_2$-), 5.90 (d, 2H, *J* =13.0 Hz, CH=CH), 7.35 (t, 2H, *J* =8.4 Hz, ArH), 7.52-7.54 (m, 4H, ArH), 7.72 (d, 2H, *J* =13.0 Hz, CH=CH), 7.91 (d, 2H, *J* =8.4 Hz, ArH), 7.93 (d, 2H, *J* =8.4 Hz, ArH), 8.11 (d, 2H, *J* =8.4 Hz, ArH), 8.40 (s, NH). ^{13}C NMR (150 MHz, DMSO-d_6) δ (ppm): 169.61, 168.94, 141.55, 138.37, 131.07, 130.92, 130.34,

130.11, 128.33, 127.78, 124.07, 122.23, 120.55, 111.93, 95.20, 71.70, 70.89, 70.33, 70.14, 67.97, 58.48, 49.47, 48.99, 47.95, 43.73, 30.44, 28.26, 25.37, 21.86. HRMS: calculated M_r = 911.5430 for $C_{55}H_{71}N_6O_6^+$, found m/z = 911.5432 ([M_r]).

Synthesis of CyP7N$_3$-BCN. CyP7N$_3$ (10 mg, 12 μmol, 1 eq.) and BCN-NHS (4 mg, 18 μmol, 1.2 eq.) were dissolved in 2 mL DMSO and the mixture was further stirred at room temperature for 0.5 h. After completion of the reaction, the mixture was washed with ether (5 mL × 3) three times to obtain the crude product. The solid was lyophilized using a freeze-dryer to give CyP7N$_3$-BCN as a blue semisolid (13 mg, 97%). ^{1}H NMR (600 MHz, DMSO-d_6): 1.57-1.66 (m, 5H, CH_2-, CH), 1.78 (t, 2H, J =6.0 Hz, CH_2-), 1.88 (s, 12H, CH_3CCH_3), 2.04-2.08 (m, 2H, CH_2-), 2.22-2.25 (m, 1H, CH), 2.31 (t, 2H, J =6.0 Hz, CH_2-), 2.53 (t, 4H, J =6.0 Hz, CH_2-), 2.73-2.76 (m, 2H, CH_2-) 3.00-3.03 (m, 3H, CH_2-, CH), 3.11 (s, 6H, CH_3), 3.23 (t, 4H, J =6.0 Hz, CH_2-), 3.35-3.36 (m, 4H, CH_2-), 3.42 (t, 4H, J =6.0 Hz, CH_2-), 3.52 (t, 4H, J =6.0 Hz, CH_2-), 3.75 (d, 2H, J =6.0 Hz, CH_2-), 3.80 (t, 4H, J =6.0 Hz, CH_2-), 4.29 (s, 4H, CH_2-), 4.43 (t, 2H, J =6.0 Hz, CH_2-), 4.47-4.53 (m, 4H, CH_2-), 5.92 (d, 2H, J = 13.2 Hz), 7.40 (t, 2H, J = 8.4 Hz), 7.57 (t, 4H, J = 9.1 Hz), 7.75 (d, 2H, J = 13.2 Hz), 7.95 (d, 2H, J = 8.4 Hz), 7.97 (d, 2H, J = 8.4 Hz), 8.14 (d, 2H, J = 8.4 Hz), 8.41 (s, NH). ^{13}C NMR (150 MHz, DMSO-d_6) δ (ppm): 170.46, 169.63, 168.99, 151.86, 144.07, 141.55, 138.34, 133.38, 131.09, 130.92, 130.34, 130.08, 129.01, 128.31, 127.75, 124.08, 122.19, 120.72, 111.93, 99.45, 95.19, 71.69,

70.89, 70.32, 70.13, 67.97, 58.48, 49.47, 47.72, 45.22, 43.76, 31.07, 28.95, 28.31, 25.89, 25.36, 22.53, 22.14, 21.78, 21.59, 21.27, 20.72, 20.28, 19.96, 17.45, 17.16. HRMS: calculated M_r = 1202.6536 for $C_{70}H_{88}N_7O_{11}^+$, found m/z = 1202.6536 ([M_r]).

Theoretical Calculation. All calculations, including the geometry optimization and molecular orbitals were performed by density functional theory (DFT) calculations at B3LYP/6-31G* level. The optimized structures with minimum energies were confimed by absence of imaginary frequencies. All the above calculations were performed by the Gaussian 09 program package.[1] The atomic coordinates are obtained through the gaussian view, the plane equations of the two planes are calculated using the atomic coordinates, and the torsion angle of the two planes is obtained according to the dihedral angle formula using the following link https://onlinemschool.com/math/assistance/cartesian_coordinate/plane_angl/ (Source data are provided as a Source Data file)

Molecular Dynamic Simulation Methodology. Molecular dynamic simulations were performed by using Gromacs-2019.5 software[2] based on an all-atomic OPLS-AA force field. Parameters and charges of organic dyes were obtained from LigParGen Server[3]. Four cyanine molecules were randomly put into a simulation box (10×10×10 nm) by using the Packmol program[4]. The system was solvated with water molecules and neutralized with Cl^- ions using gmx command toolkit. Afterward, the energy of the system was minimized until lower than 500 kJ mol^{-1}. The steepest-descent method was applied to eliminate unreasonable contacts during the minimization step. Then, the equilibrium step under NVT canonical ensemble was performed (constant number of atoms, temperature and volume) at 300 K (V-rescale thermal bath) for 5 ps, followed by a NPT canonical ensemble equilibrium step (constant number of atoms, temperature and pressure) at 1 bar (Berendsen controller)

and 300 K (V-rescale thermal bath) for another 5 ps. Above steps were performed under position restrain regime (restrain force of 1000 kJ mol^{-1} nm^{-1} was applied for carbon atoms of the cyanine molecules). Another NPT canonical ensemble equilibrium step was performed under same condition of above NPT steps, except for the restrain force was reduced to 500 kJ mol^{-1} nm^{-1}. After the equilibrium step, 100 ns simulation was carried out under NPT canonical ensemble. VMD (version 1.9.4a51)[5] and Open-Source PyMOL (version 2.5.0, Schrodinger, LLC.) software were used for trajectory surveillance and visualization.

Fluorescence quantum yield. Fluorescence quantum yields (Φ) of cyanines were determined relative to a standard solution of ICG in water (Φ_F = 0.043 at room temperature). The quantum yields were calculated according to the following Equation (1):

$$\Phi_X = \Phi_S \left(\frac{A_S}{A_X}\right)\left(\frac{I_X}{I_S}\right)\left(\frac{n_X}{n_S}\right)^2 \quad (1)$$

where Φ is the fluorescence quantum yield, A is the absorbance at the excitation wavelength, I is the integrated emission intensities, n is the refractive index of the solution, and the subscripts x and s refer to the test sample and the standard sample, respectively.

Click kinetics. The second-order rate constant of the reaction between methyltetrazine and TCO was determined by the disappearance of the maxima absorbance (520 nm) of methyltetrazine-amine. In our work, to eliminate the interference of tail absorption from 680 nm of CyP7T, the absorbance at 490 nm was measured using a microplate reader (Promega GloMax Discover system). The concentration of CyP7T used was 1×10^{-4} M, and the concentrations of TCO-NHS used were 7.9×10^{-4} M, 12.6×10^{-4} M and 25.2×10^{-4} M. All the stock solutions were prepared in DMSO. The second-order rate constant (k_2) was determined under pseudo-first-order conditions. First, the absorbance (490 nm) vs time from all three independent experiments was plotted by exponential fit to obtain k_{obs}. Then, k_2 was calculated from the slope

of the plotted of k_{obs} versus three TCO concentrations. The second order rate constant was confirmed by nonlinear fitting of the absorbance vs time curves to the second order rate equation (2) [6, 7]:

$$[A] = \frac{\varepsilon([CyP7T_0][TCO_0]-[TCO_0]^2)}{[TCO_0]e^{([TCO_0]-[CyP7T_0])kt}-[TCO_0]} \quad (2)$$

where ε is the extinction coefficient of CyP7T at 490 nm, $CyP7T_0$ and TCO_0 is the experimental concentrations, A is the absorbance at 490 nm, t refers to seconds and k refers to k_2.

Cytotoxicity. To determine the cytotoxicity of CyP7T and CyP7TT, 4T1 cells (5×10^3 cells/well) were seeded in 96-well plates and incubated overnight. For CyP7T group, the cells were incubated with CyP7T at concentrations of 0, 1, 1.5, 2, 2.5 or 5 μM for 12 h or 24 h at 37 °C. For CyP7TT group, the cells were pretreated with TCO-NHS at concentration of 0, 2, 3, 4, 5 or 10 μM for 3 h at 37 °C, then incubated with CyP7T at concentrations of 0, 1, 1.5, 2, 2.5 or 5 μM for 12 h or 24 h at 37 °C, washed with PBS. Next, 10 μL of CCK-8 agent was added per well for another 3 h at 37 °C. The absorbance of each well at 450 nm was recorded with a multi-well plate reader (Promega Glomax discover system) to determine the relative cell viabilities.

Cell-targeted imaging. Almost 1 × 10^5 4T1 or MCF-7 cells were plated on in a confocal chamber and incubated at 37 °C in a 5% CO_2 incubated overnight. The TCO-RGD and TCO groups were pre-incubated 10 μM TCO-RGD or TCO-NHS for 3 h at 37 °C, repectively. Then the medium was replaced for removal of excessive TCO. Next, the cells were incubated with CyP7T (5 μM) for 10 min. Then the cells were washed three times with PBS buffer to remove free dyes before imaging. The cells were fixed with 4% paraformaldehyde for 30 min, and washed 3 times using PBS. Then the nuclei were stained with DAPI (10 μg/mL) for 5 minutes, washed with PBS 3 times.

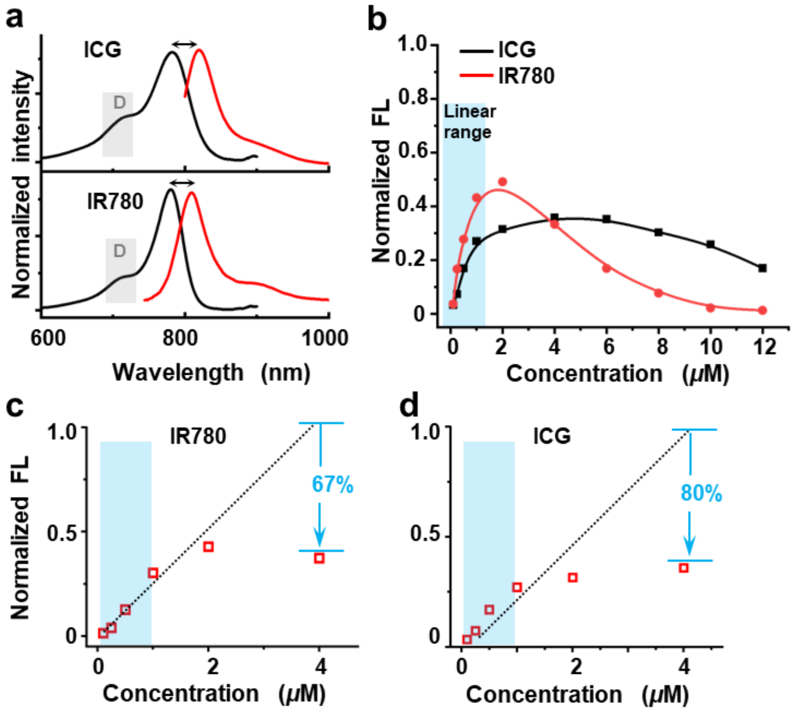

Supplementary Figure 1. Absorption and emission spectra of ICG and IR780 in methanol, D denotes H-type dimer (a). Normalized fluorescence intensity of cyanine dyes with concentrations (b). Fluorescence loss of dyes with concentrations of IR780 (c) and ICG (d). Dot lines represent ideal linear relationship between fluorescence intensity and concentration, indicating non-quenching below 1 μM.

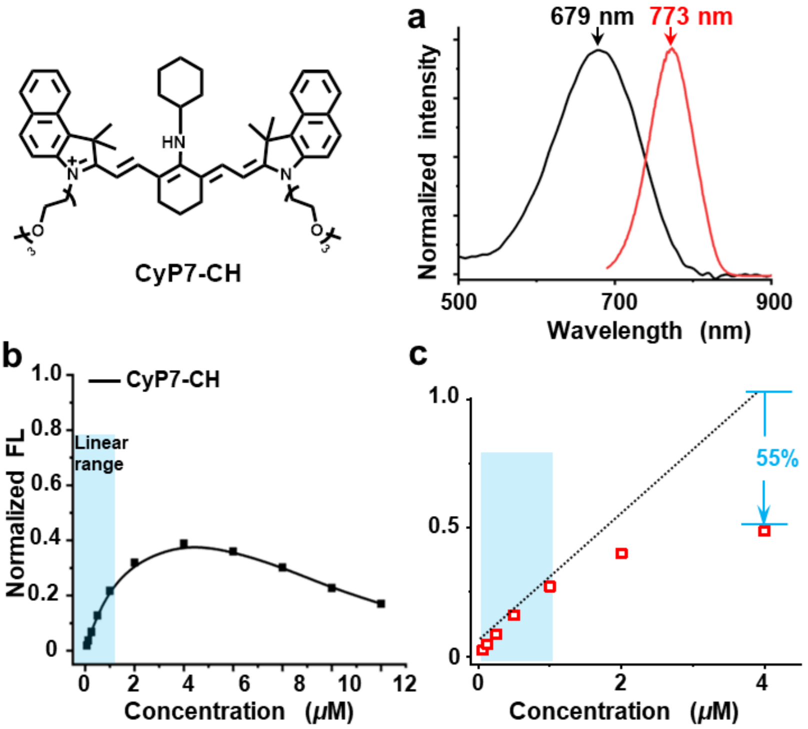

Supplementary Figure 2. Absorption and emission spectra of CyP7-CH in methanol. (a) Normalized fluorescence intensity of cyanine dyes at various concentrations. (b) Fluorescence quenching of dyes at various concentrations. (c) Dotted lines represent ideal linear relationship between fluorescence intensity and concentration, indicating non-quenching below 1 *μ*M.

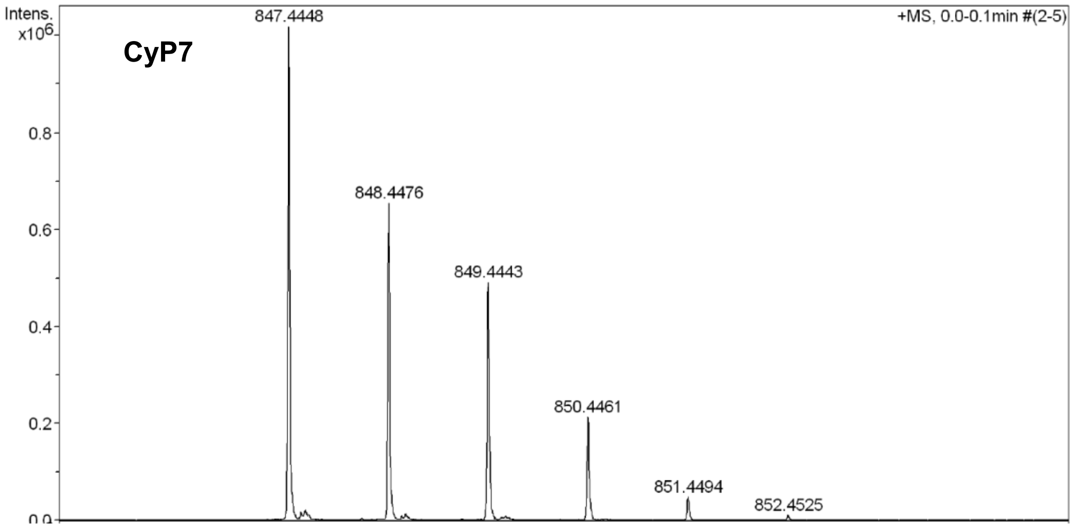

Supplementary Figure 3. High-resolution mass spectra (HRMS) of CyP7.

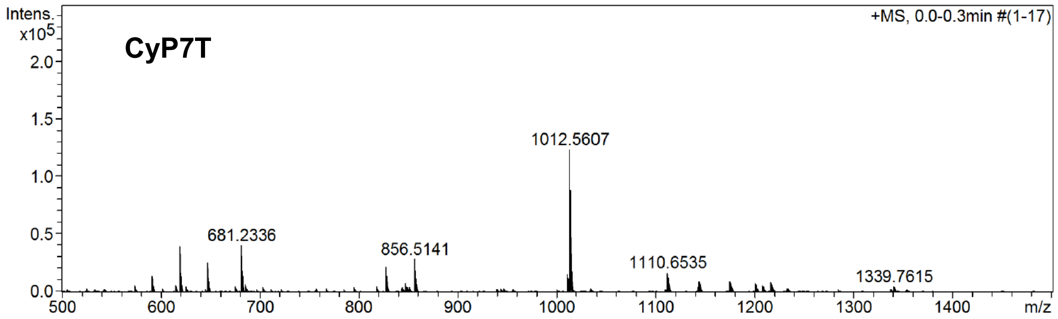

Supplementary Figure 4. High-resolution mass spectra (HRMS) of CyP7T.

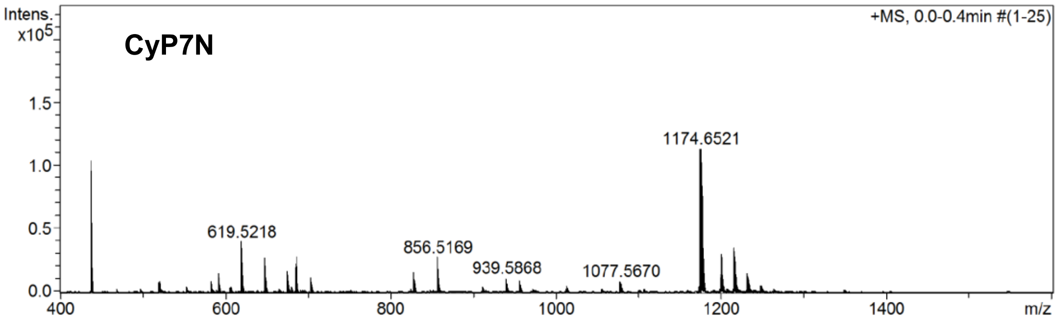

Supplementary Figure 5. High-resolution mass spectra (HRMS) of CyP7N.

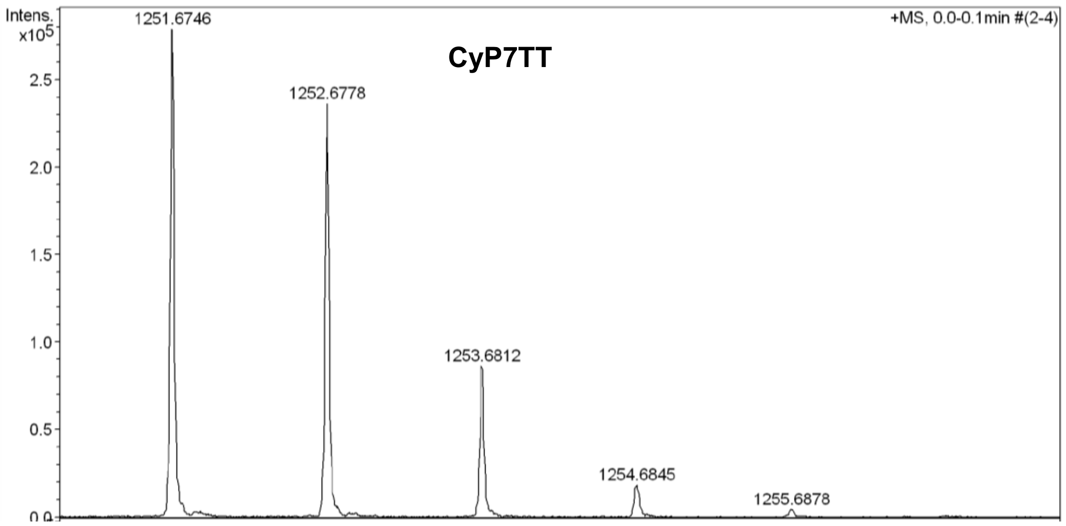

Supplementary Figure 6. High-resolution mass spectra (HRMS) of CyP7TT.

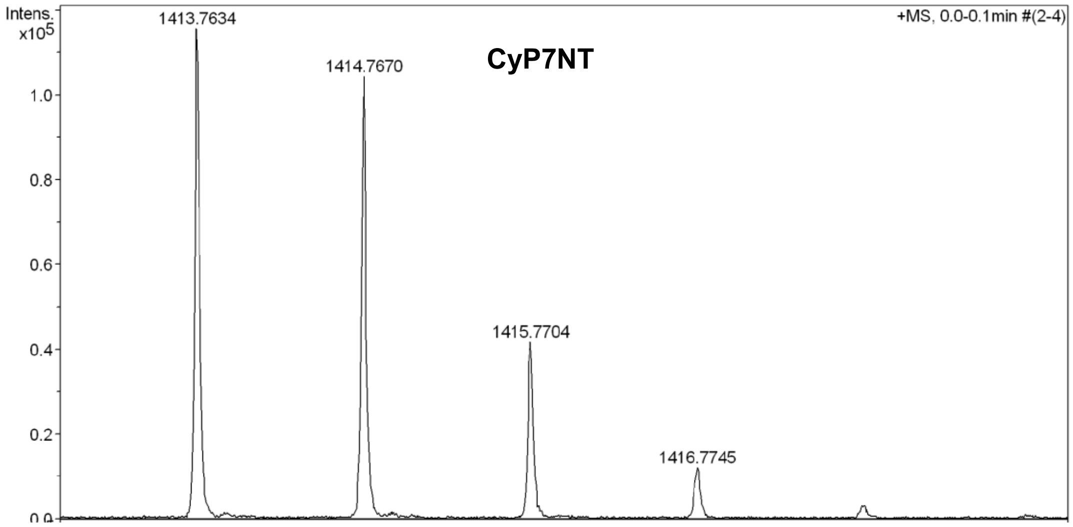

Supplementary Figure 7. High-resolution mass spectra (HRMS) of CyP7NT.

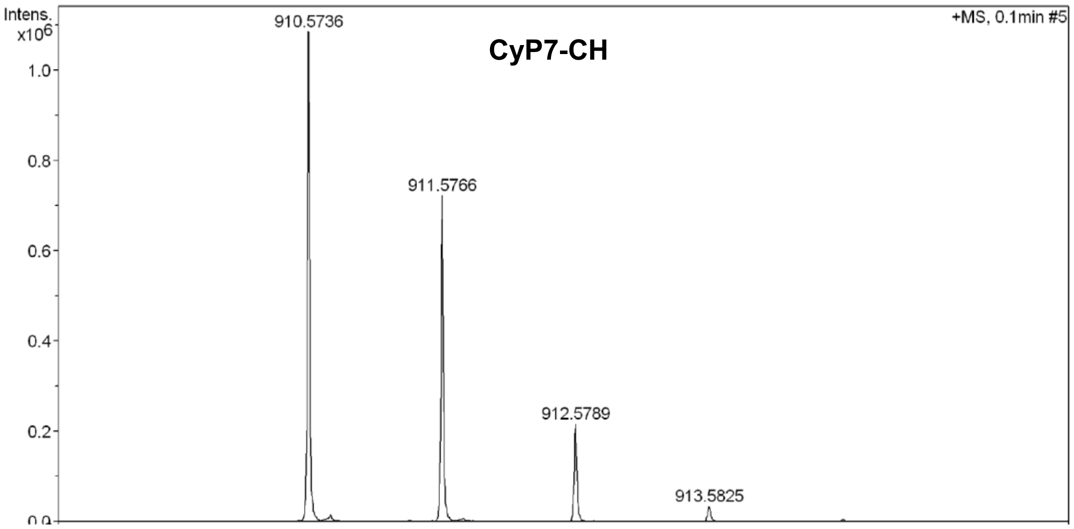

Supplementary Figure 8. High-resolution mass spectra (HRMS) of CyP7-CH.

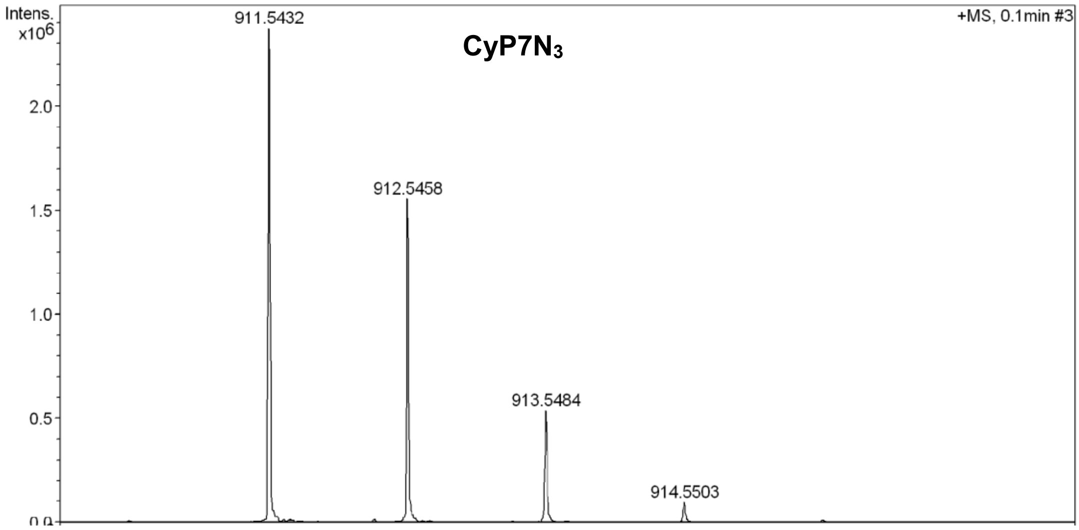

Supplementary Figure 9. High-resolution mass spectra (HRMS) of CyP7N_3.

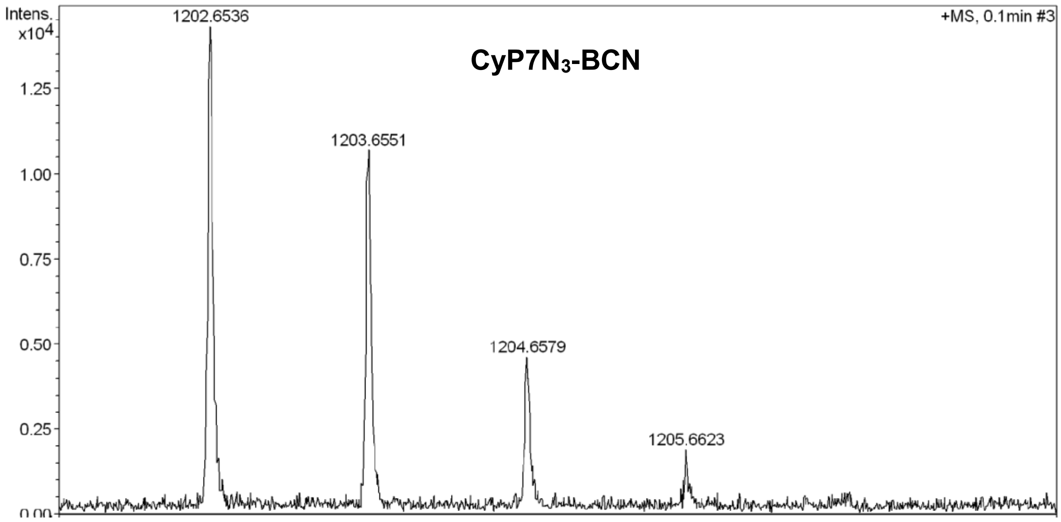

Supplementary Figure 10. High-resolution mass spectra (HRMS) of CyP7N_3-BCN.

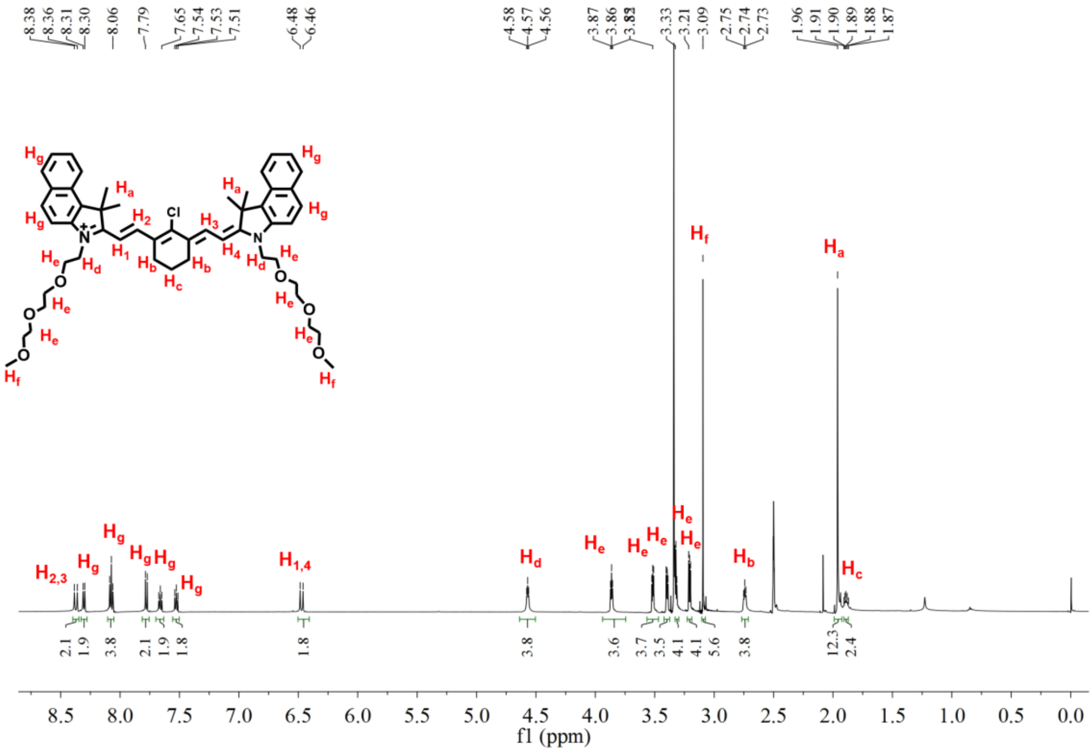

Supplementary Figure 11. ^{1}H NMR of CyP7.

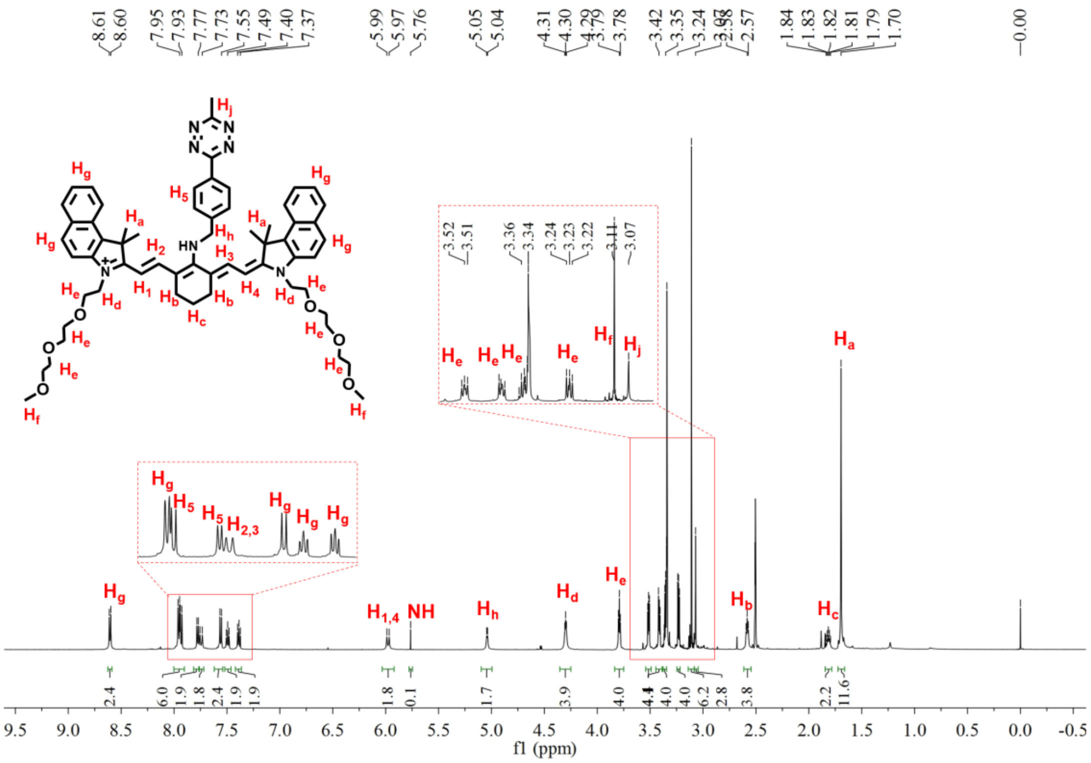

Supplementary Figure 12. ^{1}H NMR of CyP7T.

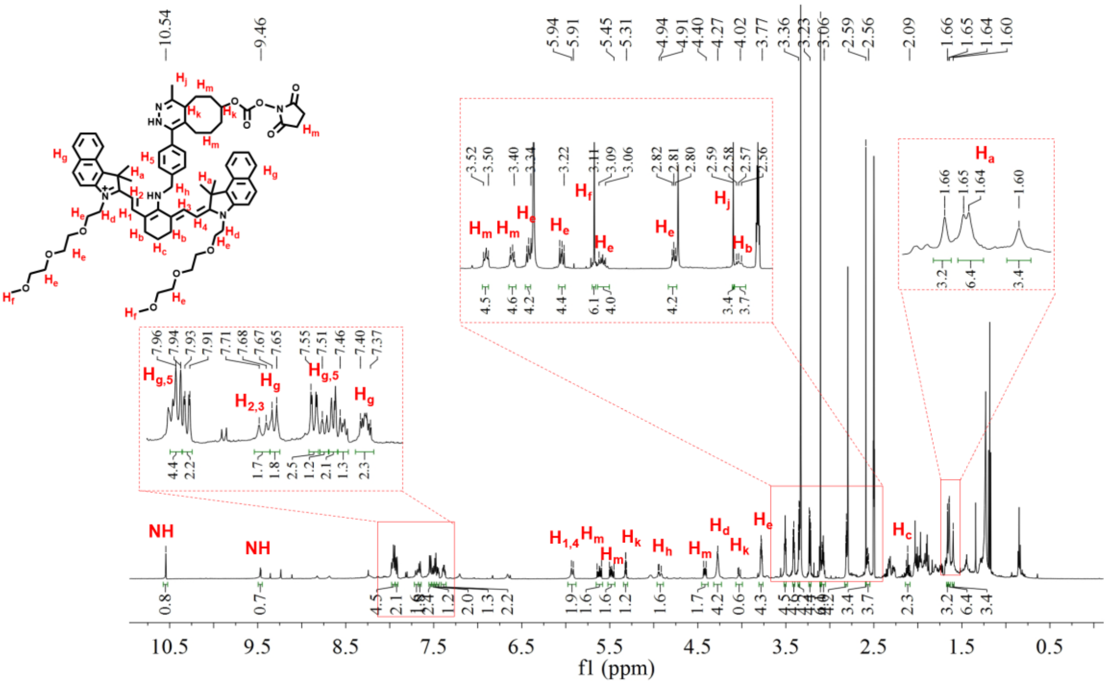

Supplementary Figure 13. ^{1}H NMR of CyP7TT.

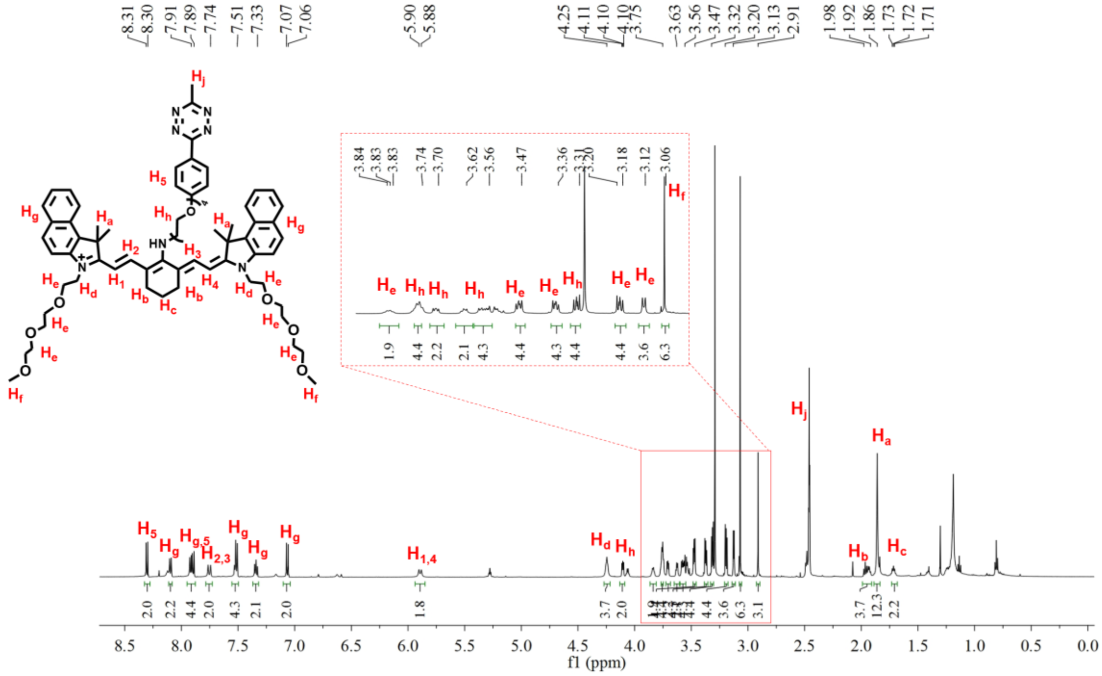

Supplementary Figure 14. ^{1}H NMR of CyP7N.

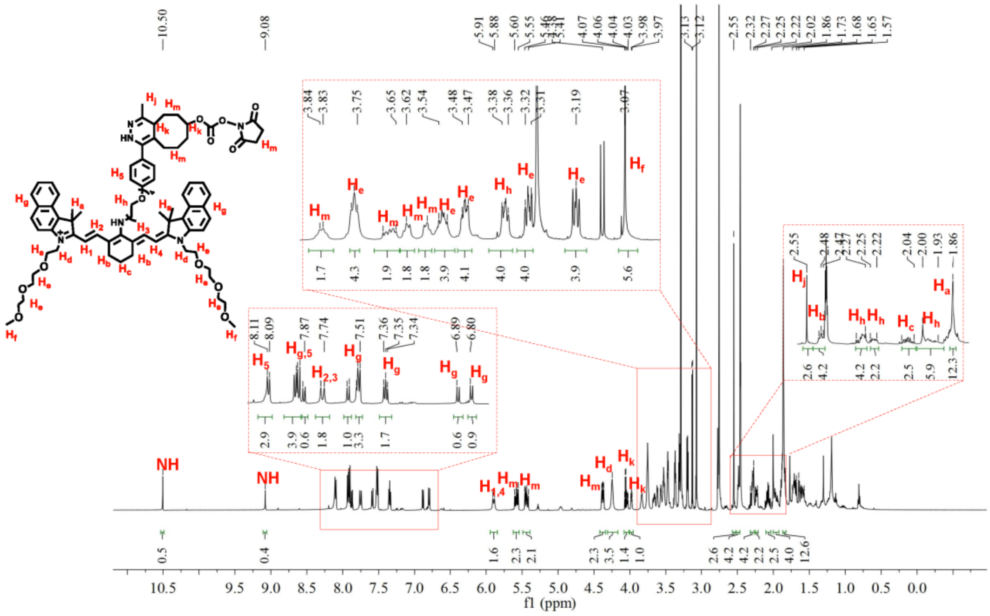

Supplementary Figure 15. ^{1}H NMR of CyP7NT.

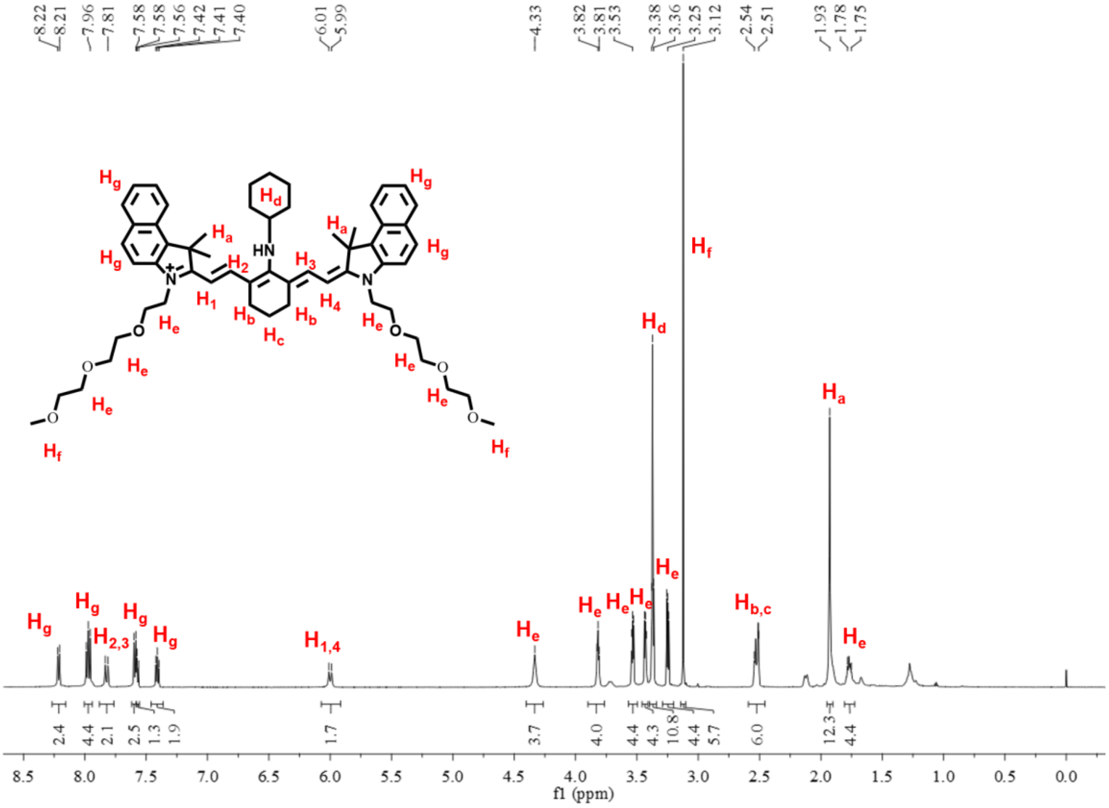

Supplementary Figure 16. ^{1}H NMR of CyP7-CH.

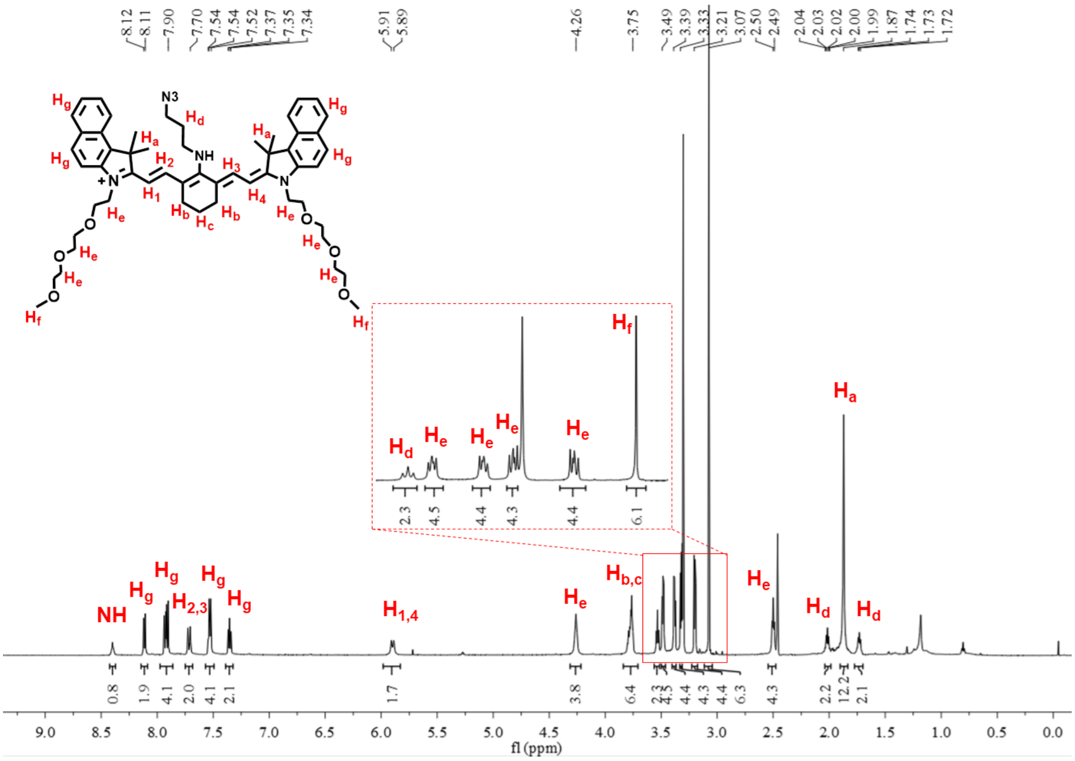

Supplementary Figure 17. ^{1}H NMR of $CyP7N_3$.

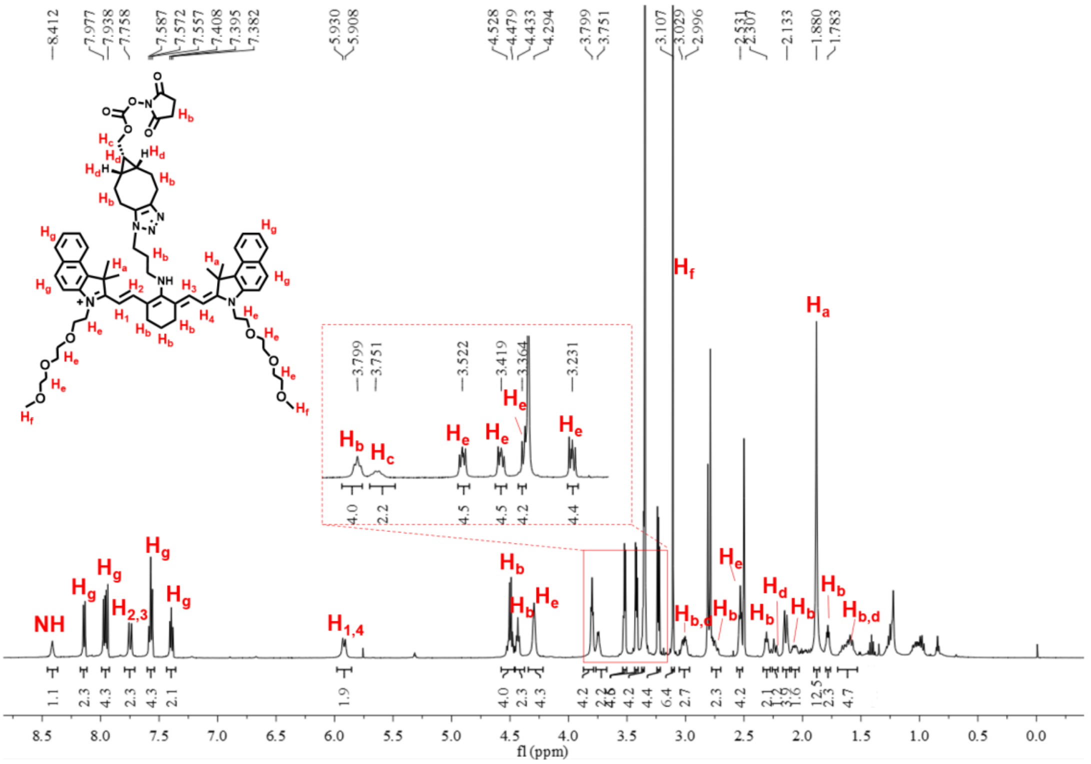

Supplementary Figure 18. ^{1}H NMR of $CyP7N_3$-BCN.

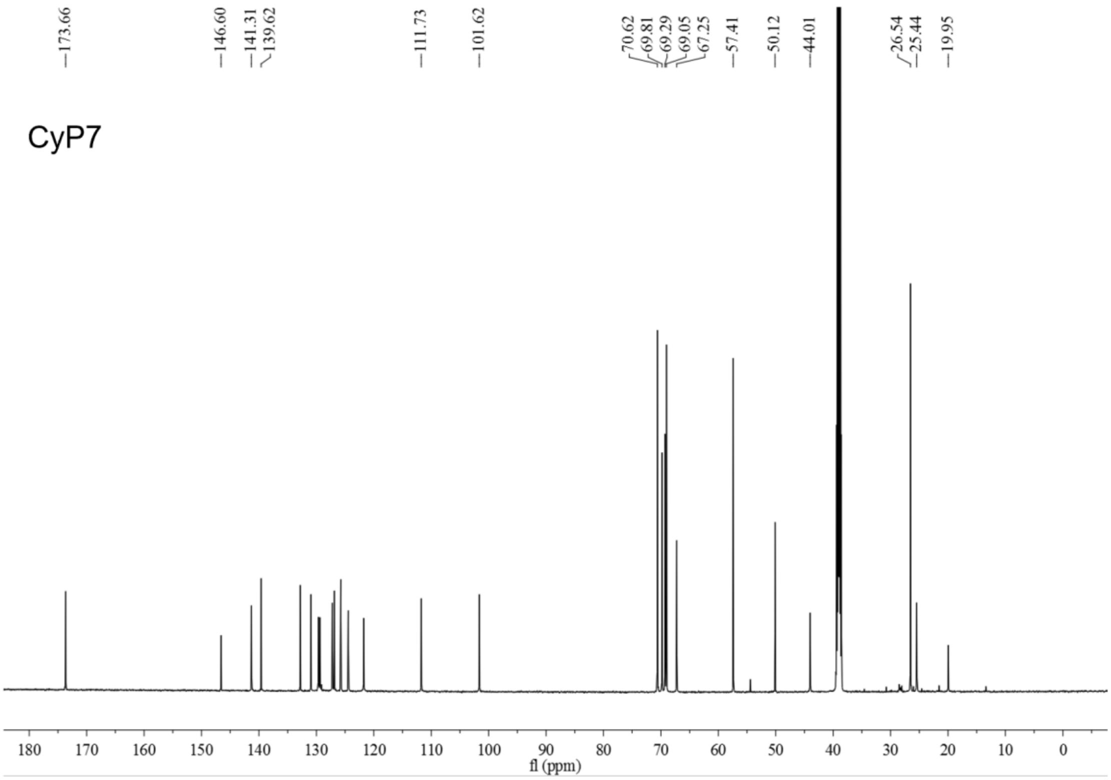

Supplementary Figure 19. ^{13}C NMR of CyP7.

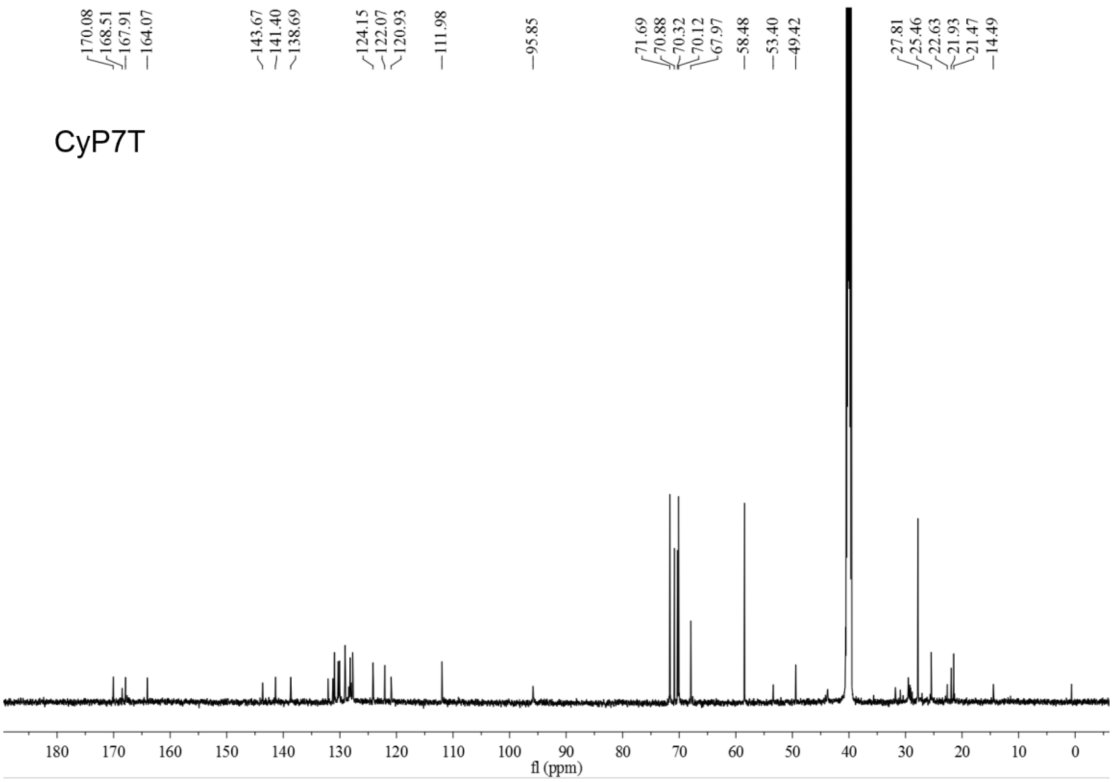

Supplementary Figure 20. ^{13}C NMR of CyP7T.

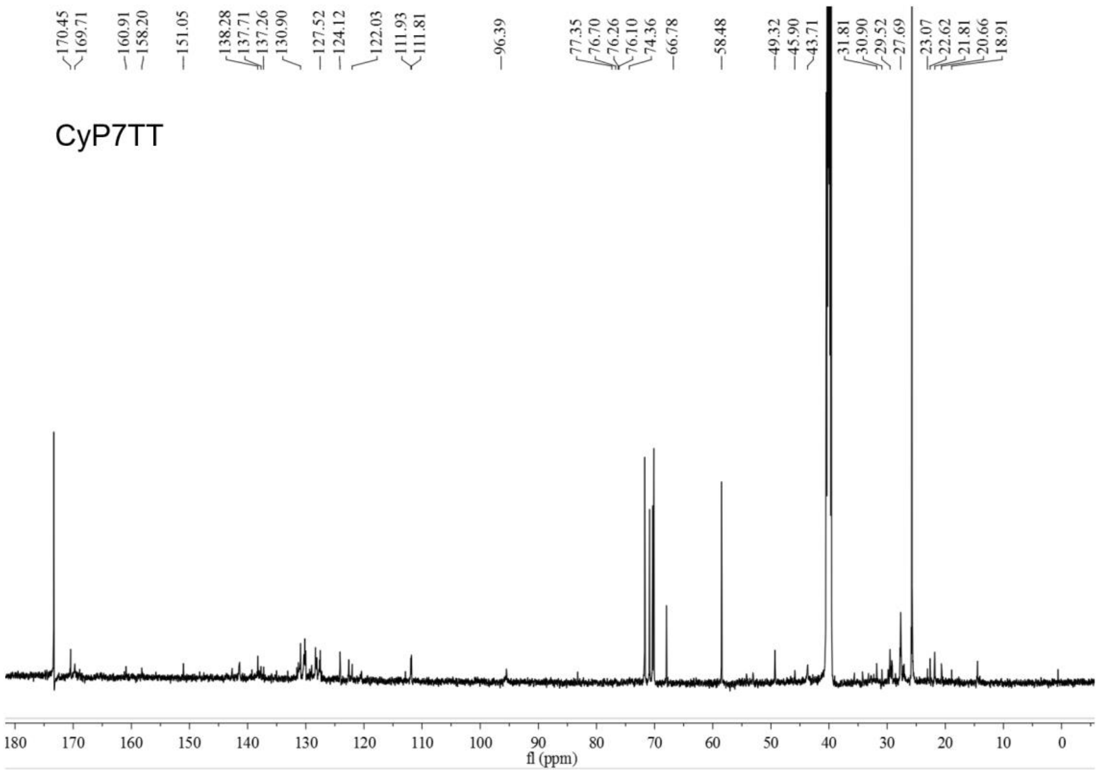

Supplementary Figure 21. ^{13}C NMR of CyP7TT.

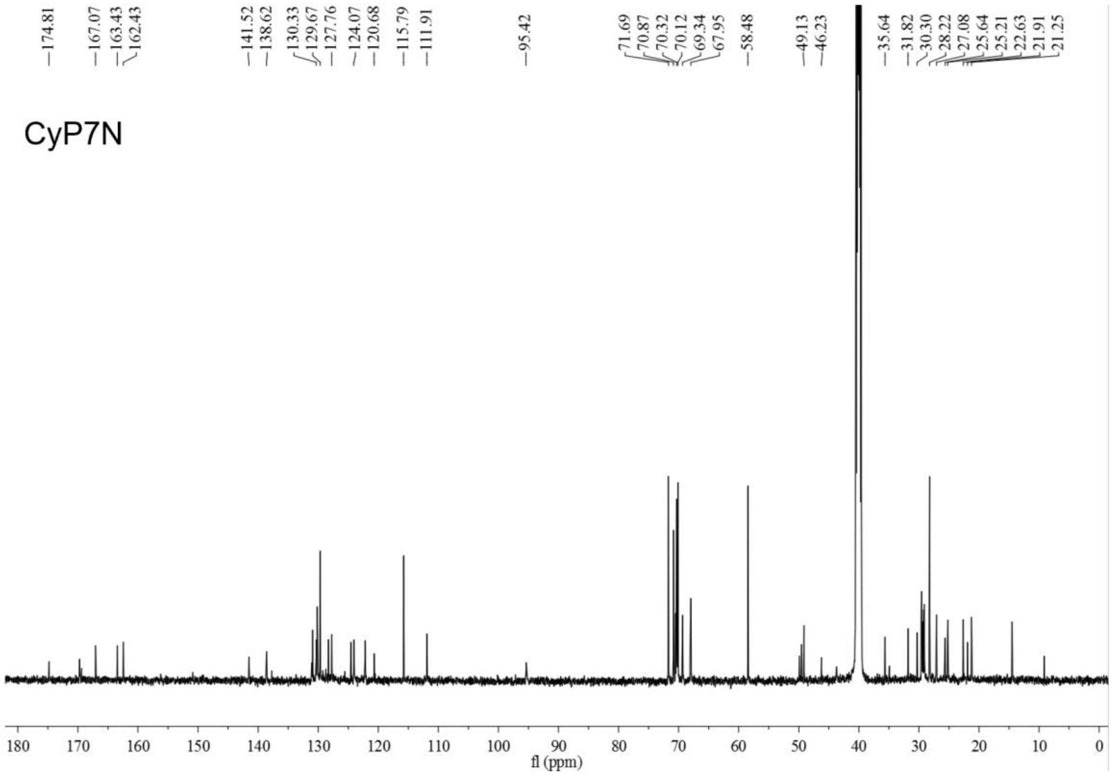

Supplementary Figure 22. ^{13}C NMR of CyP7N.

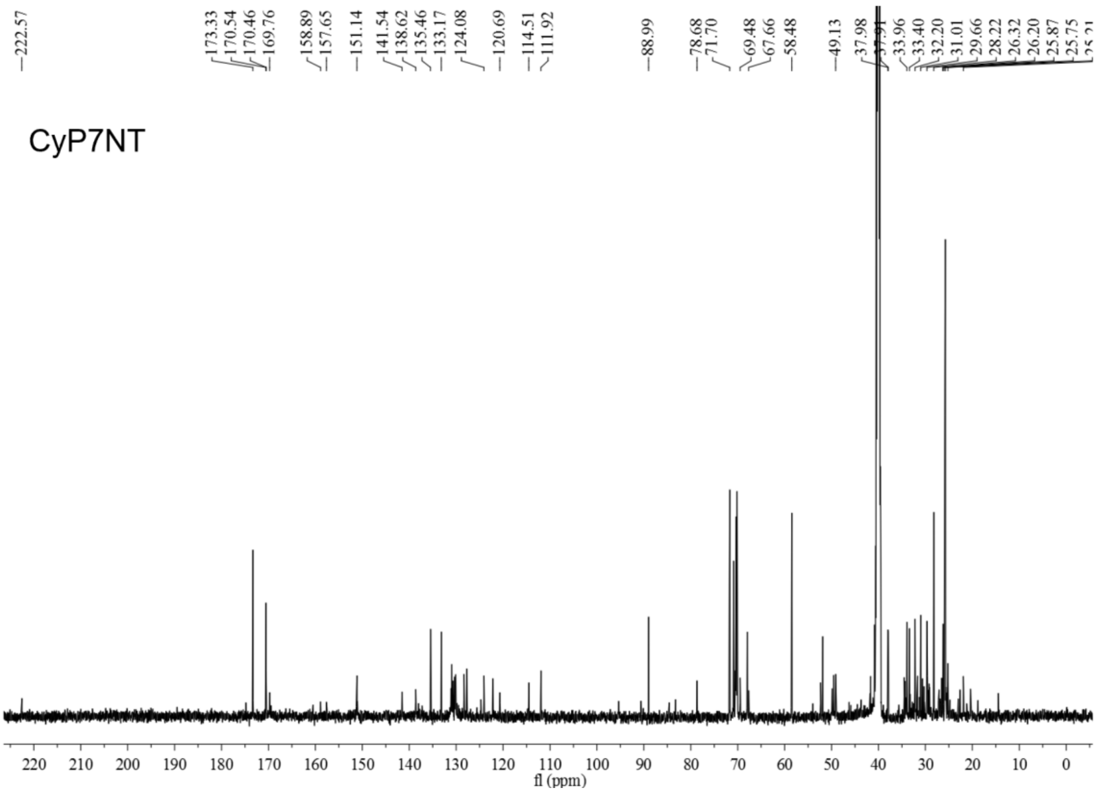

Supplementary Figure 23. ^{13}C NMR of CyP7NT.

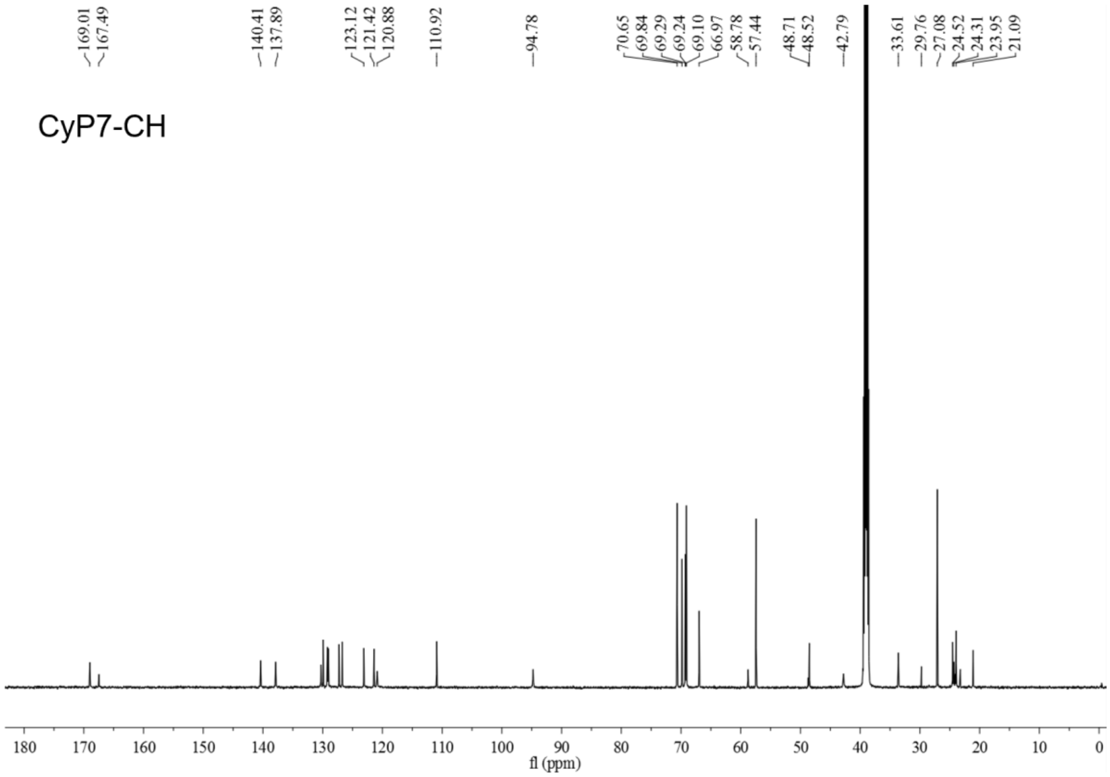

Supplementary Figure 24. ^{13}C NMR of CyP7-CH.

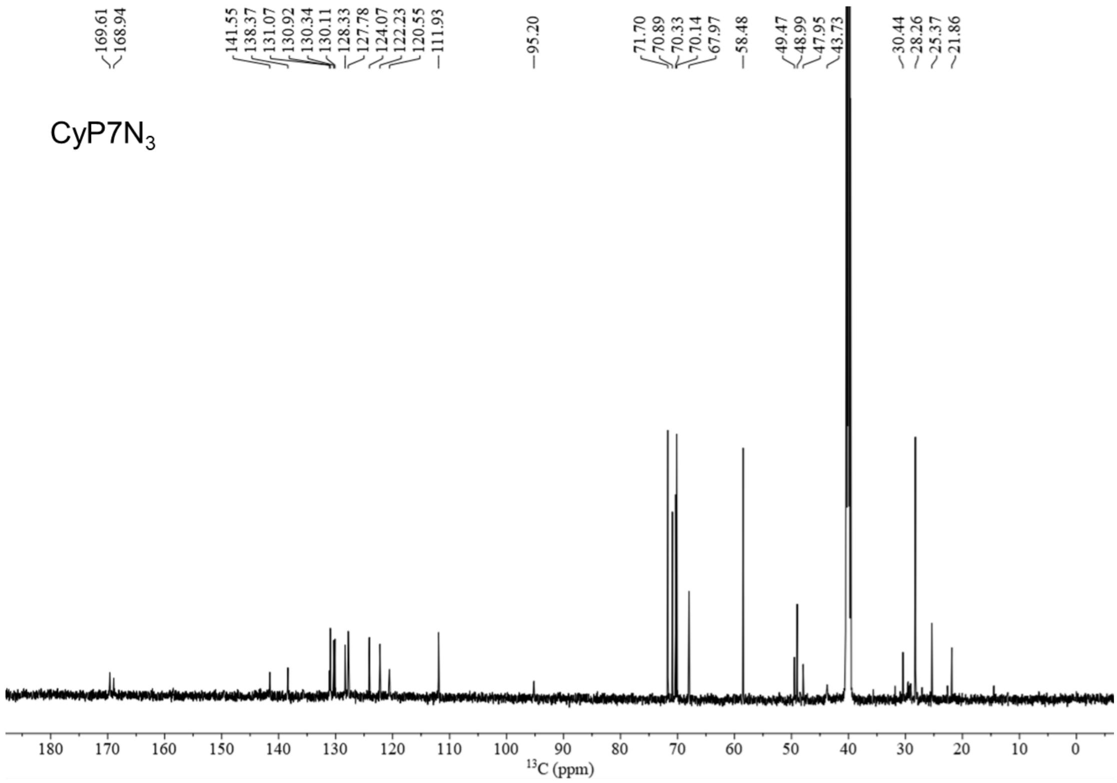

Supplementary Figure 25. ^{13}C NMR of CyP7N_3.

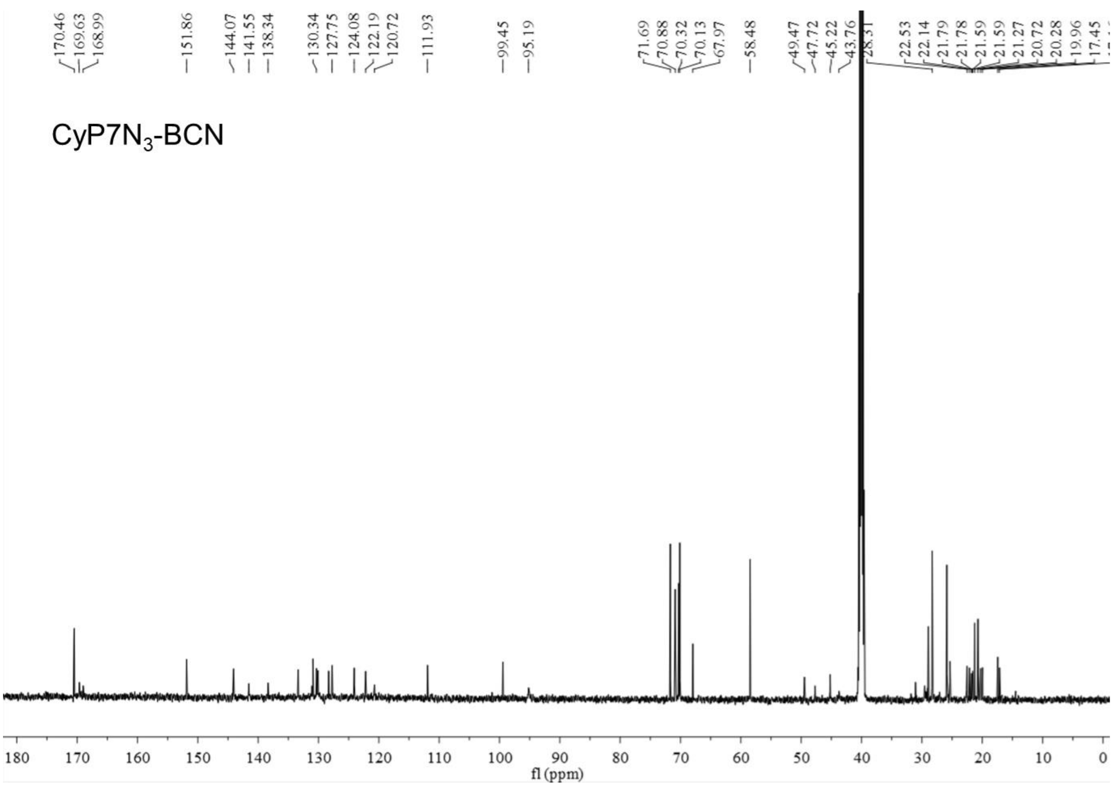

Supplementary Figure 26. ^{13}C NMR of CyP7N$_3$-BCN.

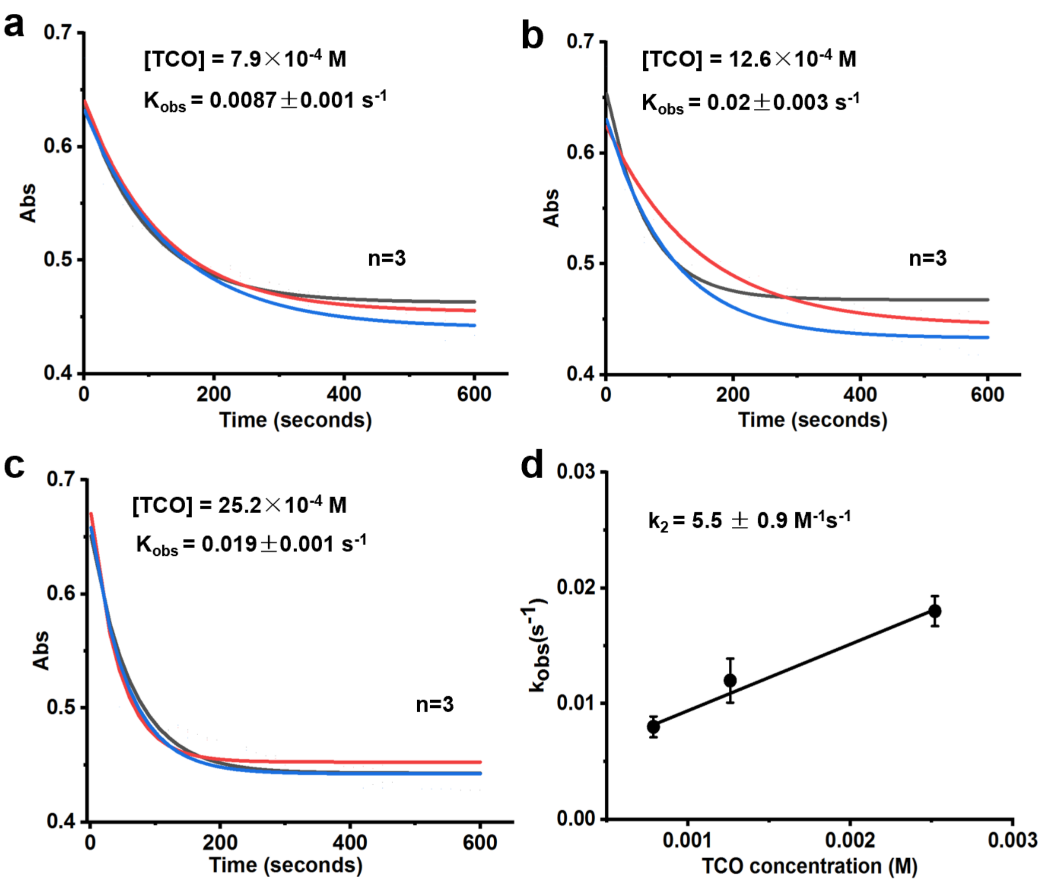

Supplementary Figure 27. Determination of second-order rate constant between methyltetrazine and TCO. (a-c) The disappearance at 490 nm was monitored over time and exponential fitted to obtain k_{obs}. (d) Different k_{obs} vs TCO concentrations were linear fitted to obtain k_2. Data are presented as mean ± SD. (n = 3 independent samples per concentration).

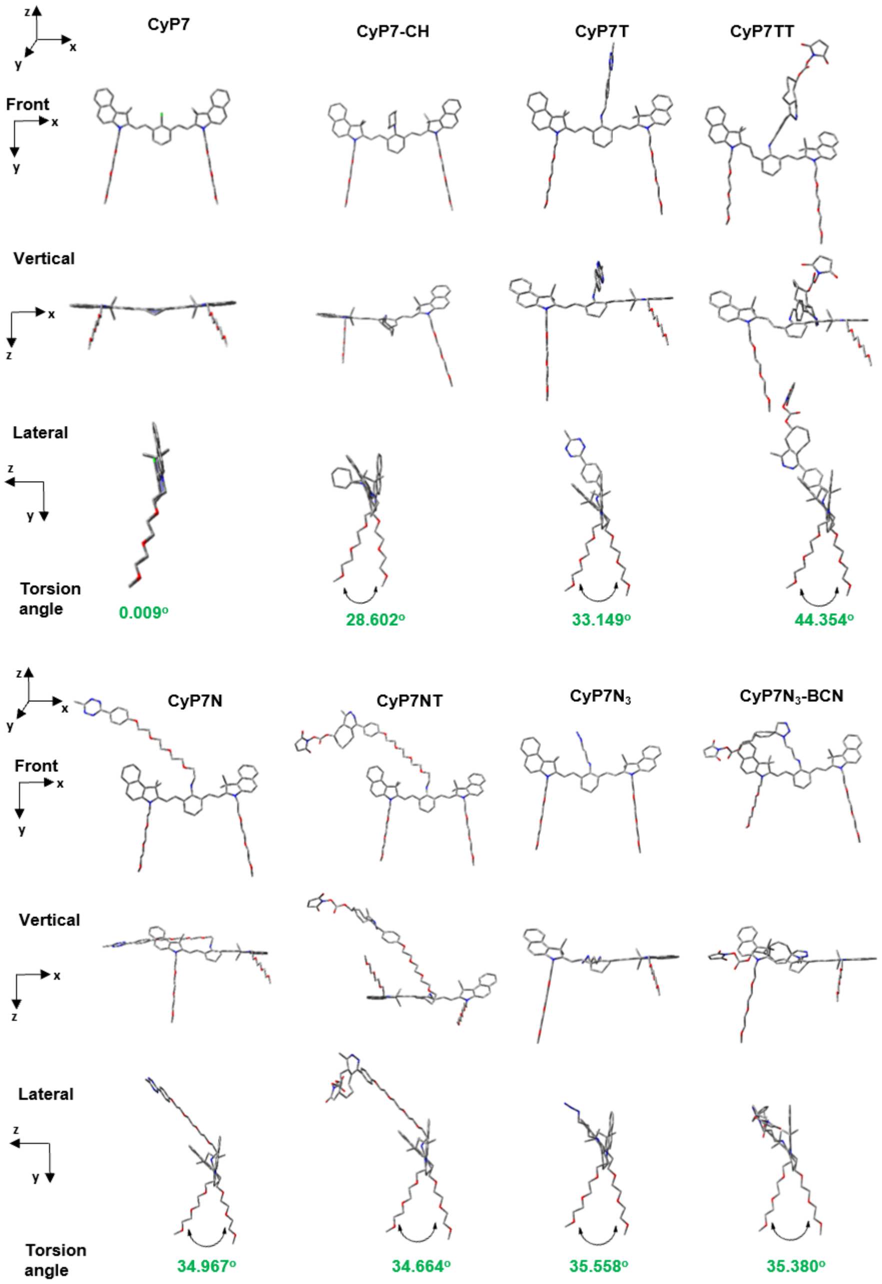

Supplementary Figure 28. Optimized molecular conformations of CyP7, CyP7-CH, CyP7T, CyP7TT, CyP7N, CyP7NT, CyP7N_3 and CyP7N_3-BCN from front, vertical and lateral views. Absence of imaginary frequencies confirmed the optimized structures with minimum energies. Torsion angles between two benzoindole rings indicated the extent of out-of-plane.

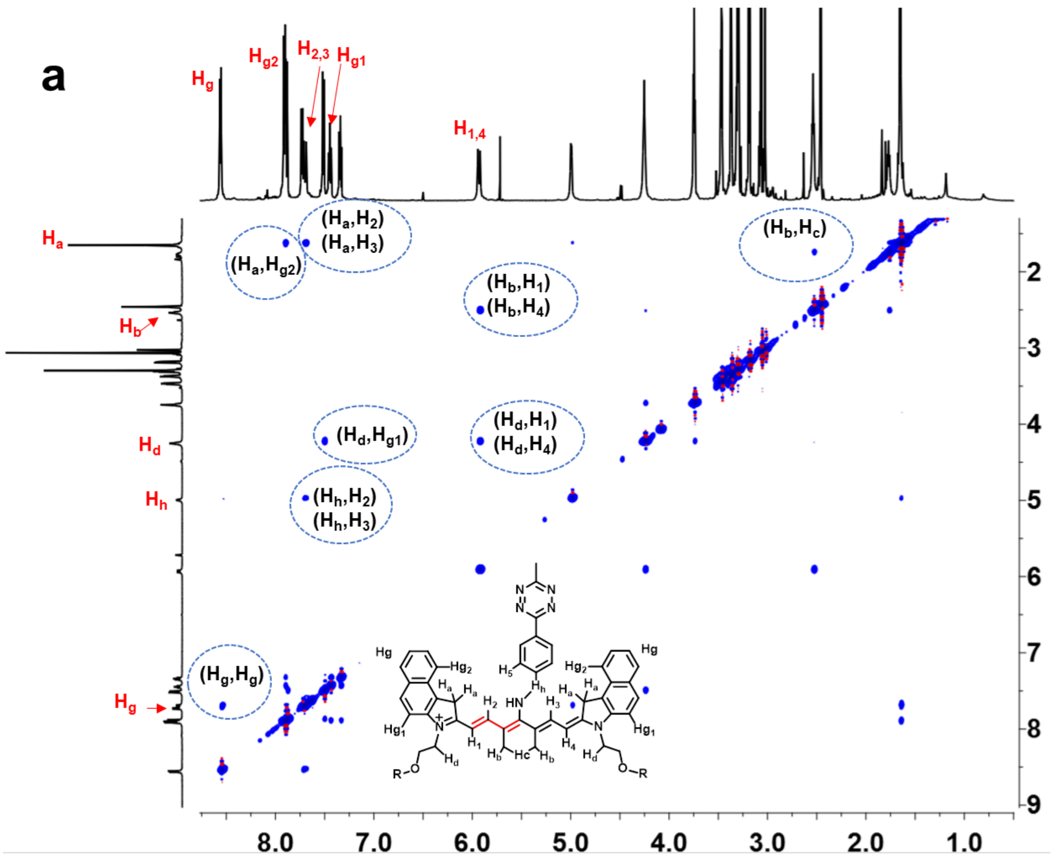

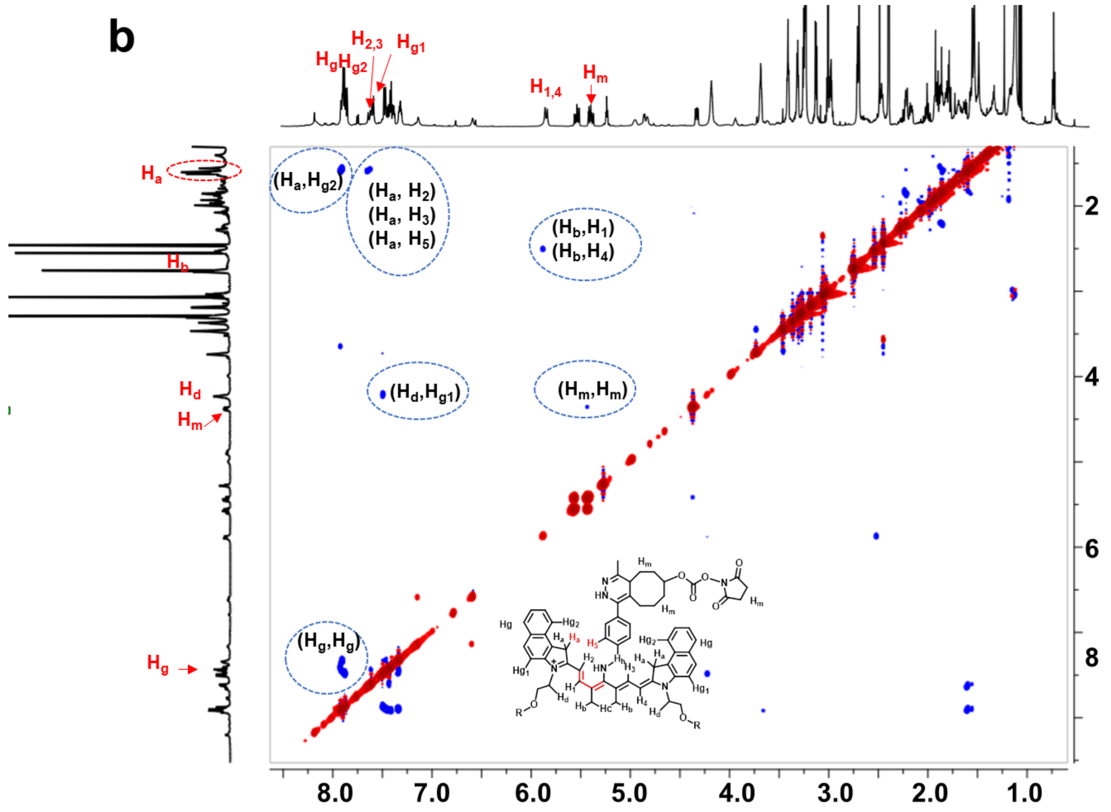

Supplementary Figure 29. 2D ROESY NMR of (a) CyP7T and (b) CyP7TT in d_6-DMSO.

a

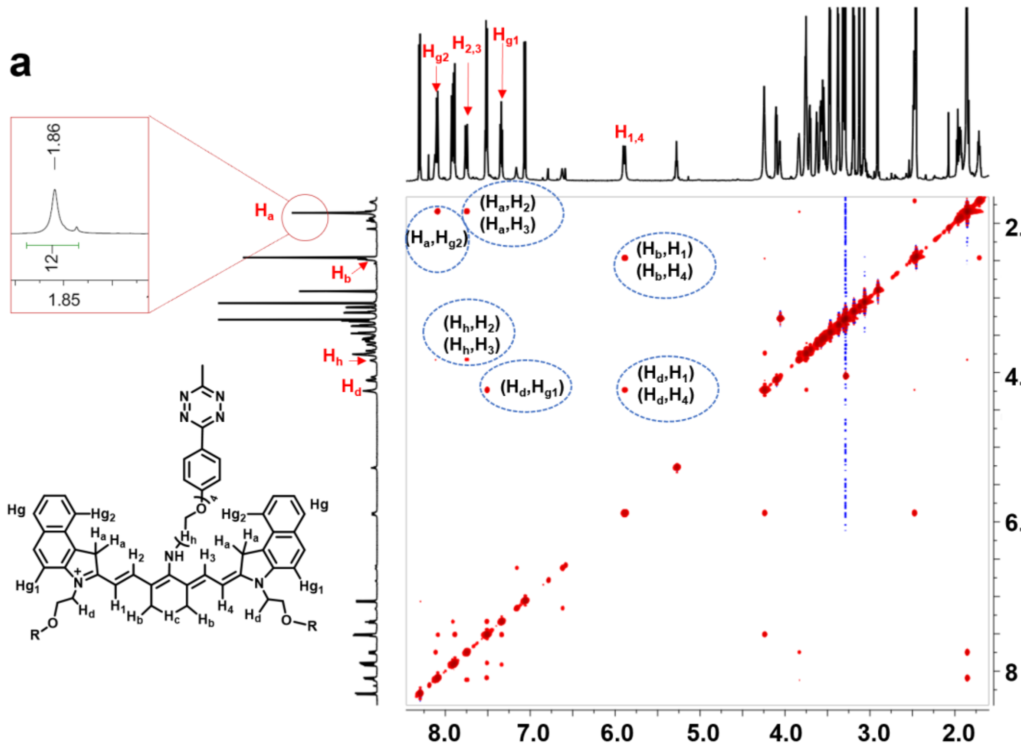

b

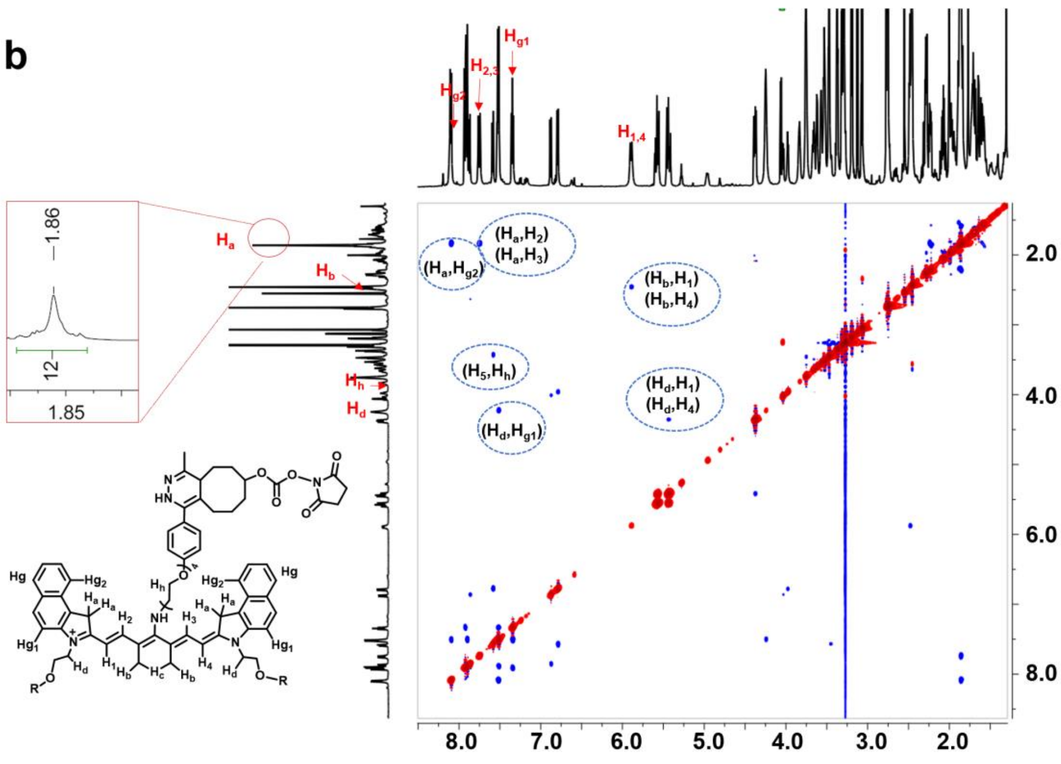

Supplementary Figure 30. 2D ROESY NMR of (a) CyP7N and (b) CyP7NT in d_6-DMSO.

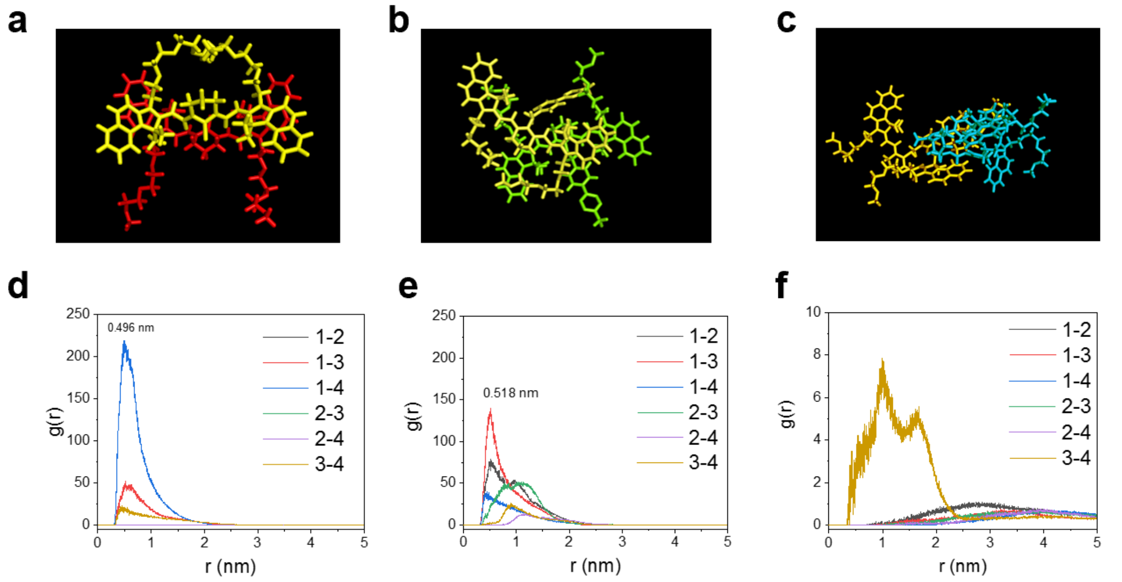

Supplementary Figure 31. Molecular dynamics simulation. Snapshot images and corresponding radial distribution functions (RDF) plots of (a,d) CyP7, (b,e) CyP7T, and (c,f) CyP7TT during 100 ns simulation period under NPT canonical ensemble condition. Four molecules were analyzed for each cyanine dye: 1-2, 1-3, 1-4, 2-3, 2-4, 3-4 represent the RDF of molecule 1 and 2, molecule 1 and 3, molecule 1 and 4, molecule 2 and 3, respectively.

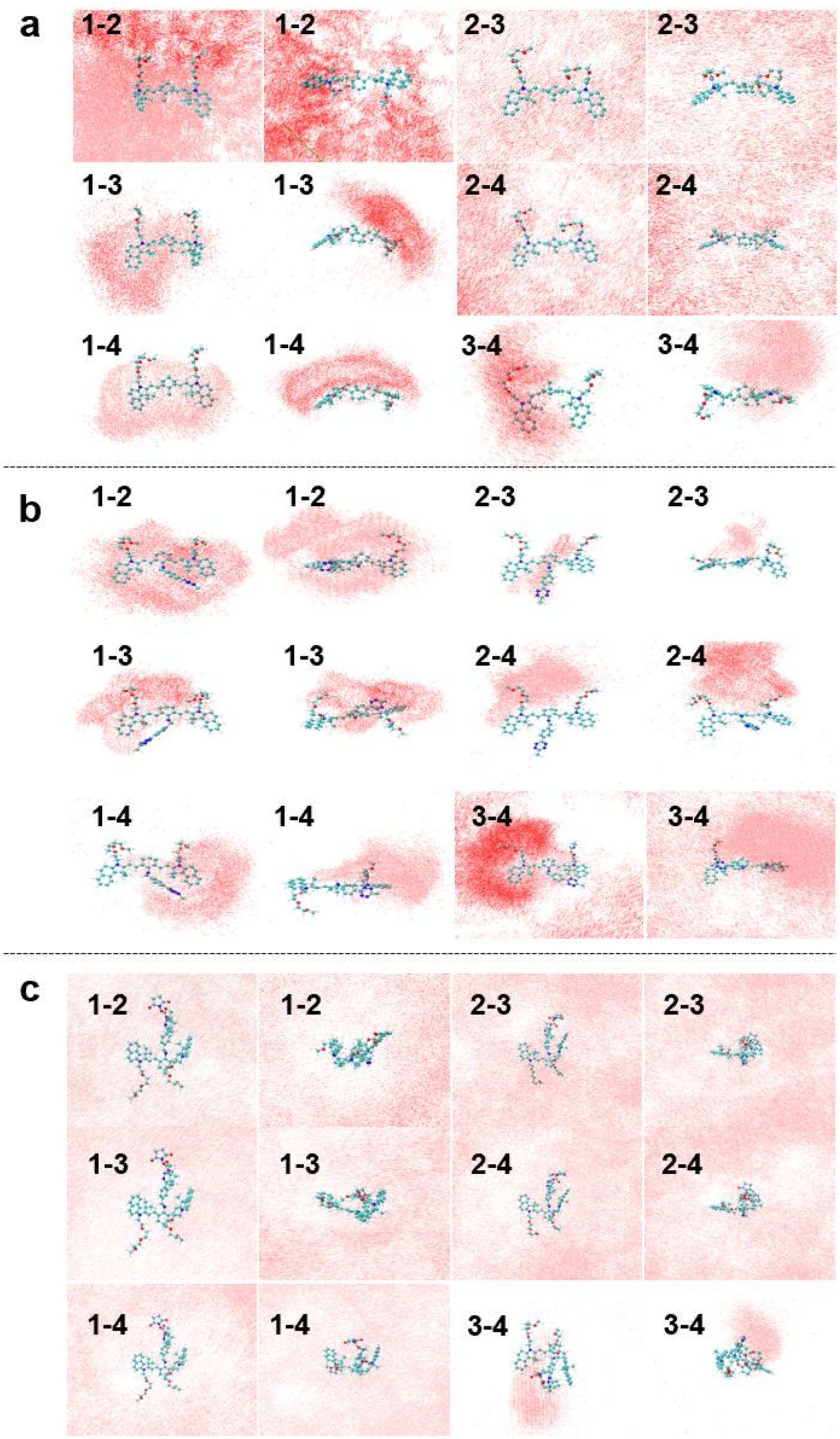

Supplementary Figure 32. Spatial distribution function (SDF) for the coordination of (a) CyP7, (b) CyP7T and (c) CyP7TT around another of the same molecule.

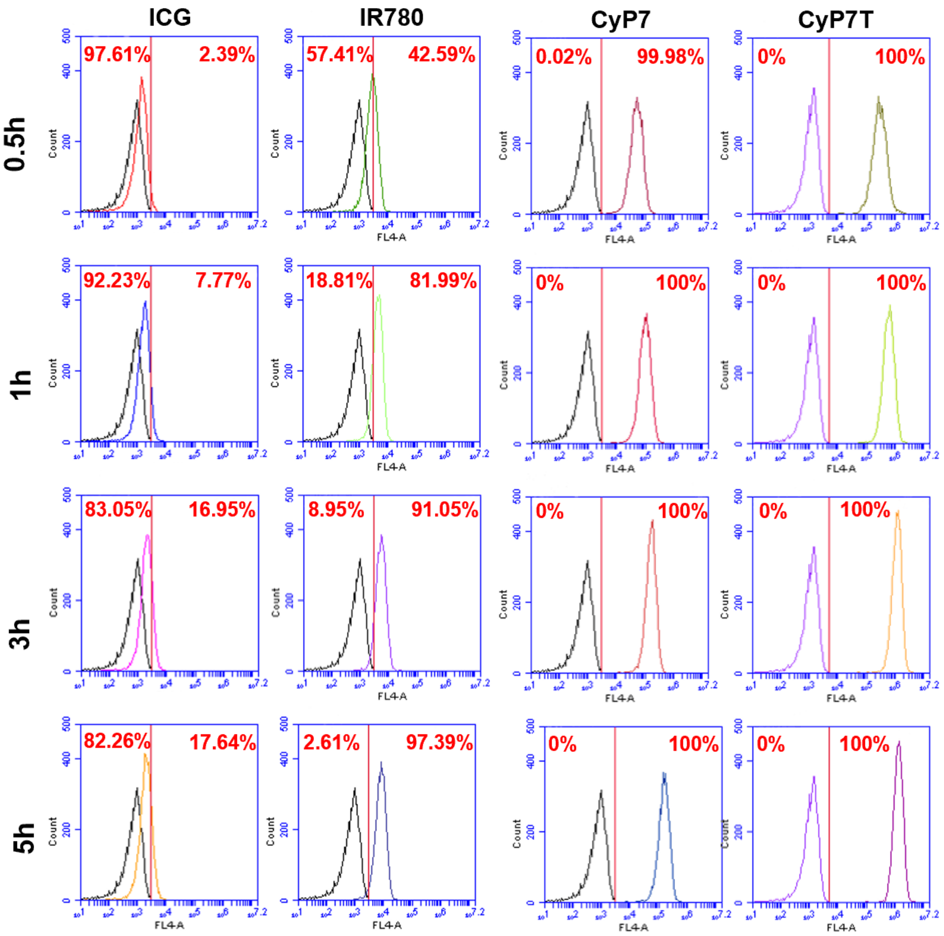

Supplementary Figure 33. Representative histograms from flow-cytometric analysis of cell permeability in 4T1 cells. Gating strategy (FSC-A/SSC-A) was used to exclude cell debris and aggregates (both for control and experiment conditions).

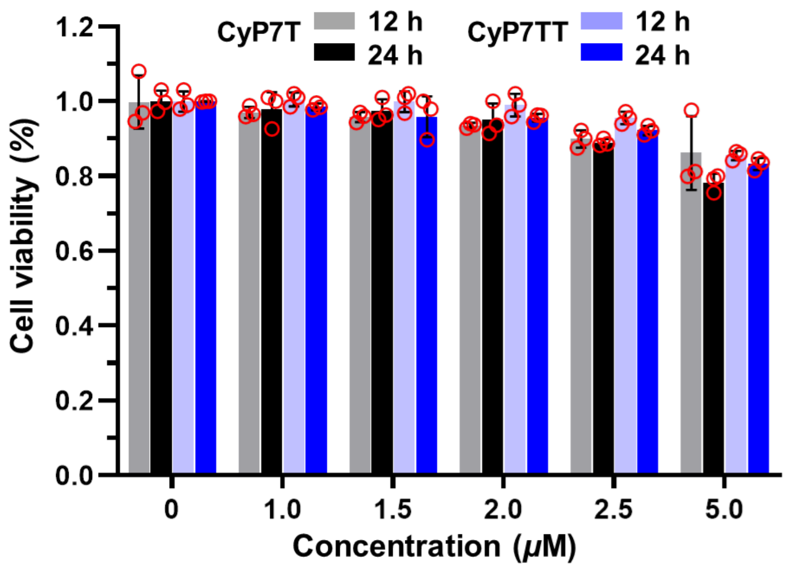

Supplementary Figure 34. Relative viabilities of 4T1 cells after incubation with CyP7T and CyP7TT (0, 1, 1.5, 2, 2.5 or 5 *μ*M) for 12 h and 24 h. Data are presented as mean ± SD. (n = 3 independent experiments per concentration).

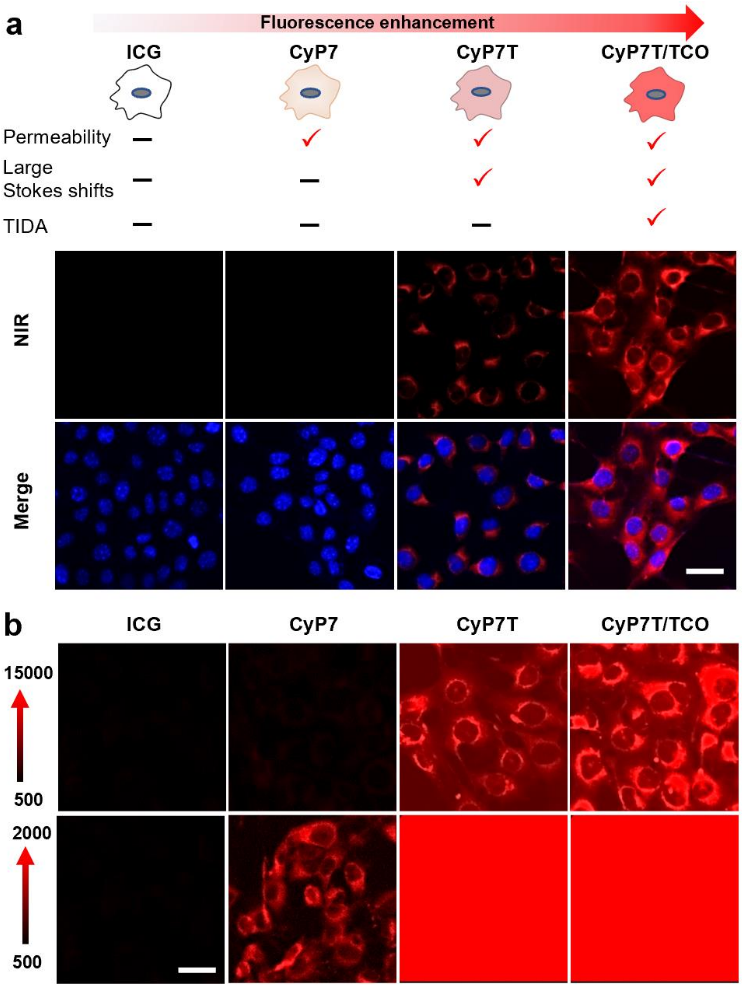

Supplementary Figure 35. Characterization of probes in 4T1 cells. (a) Fluorescence microscopy imaging of 4T1 cells labeled with ICG, CyP7, CyP7T and CyP7T/TCO (5 μM, 0.5 h). For CyP7T/TCO, cells were pre-incubated with 10 μM TCO for 3 h, washed with PBS, and then followed by incubation with 5 μM CyP7T for 0.5 h. Cell were stained with nuclear dye, DAPI (blue). (b) NIR images of ICG, CyP7, CyP7T and CyP7T/TCO (5 μM, 0.5 h) using different fluorescence intensity scale bars. n = 3 independent samples per probe. All the images were acquired at 40× magnification. Scale bar: 30 μm.

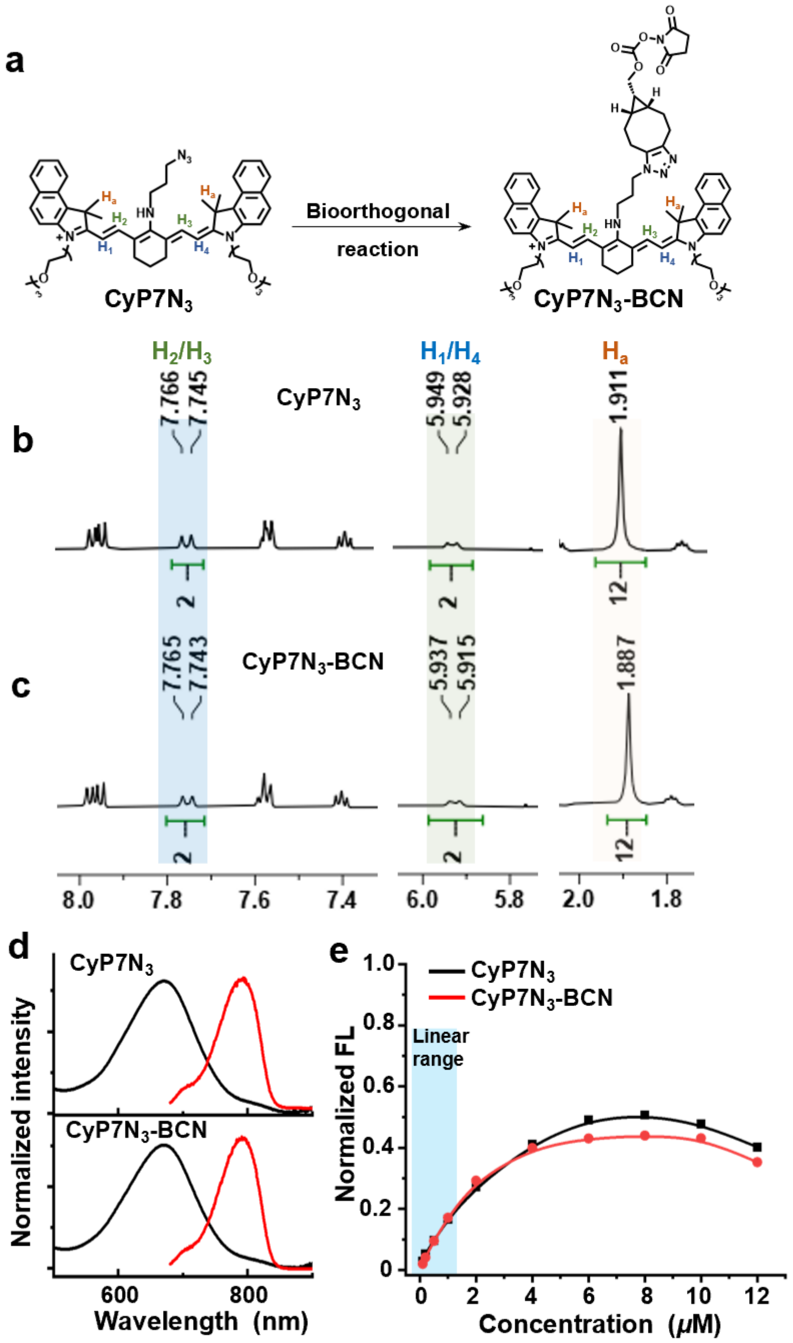

Supplementary Figure 36. Bioorthogonal negative control ligation of azide-bicyclononyne (N_3-BCN). Structures of probes CyP7N_3 and CyP7N_3-BCN (a), ^{1}H NMR shifts of vinyl-hydrogens (H_1, H_2, H_3, H_4) and $C(CH_3)_2$ protons (H_a) for CyP7N_3 (b) and CyP7N_3-BCN (c). Absorption/emission spectra (d) and normalized fluorescence intensity with concentrations (e) of CyP7N_3 and CyP7N_3-BCN.

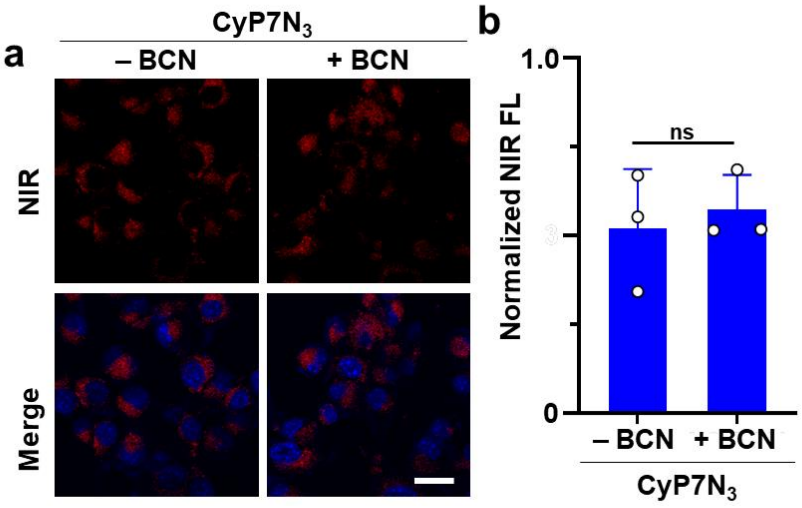

Supplementary Figure 37. CLSM bioorthogonal imaging of 4T1-cells treated with $CyP7N_3$ in response to BCN. (a) Fluorescence microscopy imaging of 4T1 cells pre-treated with 10 μM BCN-NHS (in RPMI-1640 medium) for 3 h. After which, the medium was removed, and the cell were washed with PBS buffer to remove excess BCN-NHS, followed by incubation with $CyP7N_3$ (5 μM) for 0.5 h. Cell were stained with nuclear dye, DAPI (blue). (b) Qualitative analysis of fluorescence in Supplementary Figure 37a. Scale bar: 30 μm. A two-sided student's t-test was performed (p = ,0.6685, ns: not significant). No statistically significant difference in fluorescence signals was observed for $CyP7N_3$ upon N_3-BCN ligation in live cells. Data are presented as mean ± SD. (n = 3 independent samples per concentration).

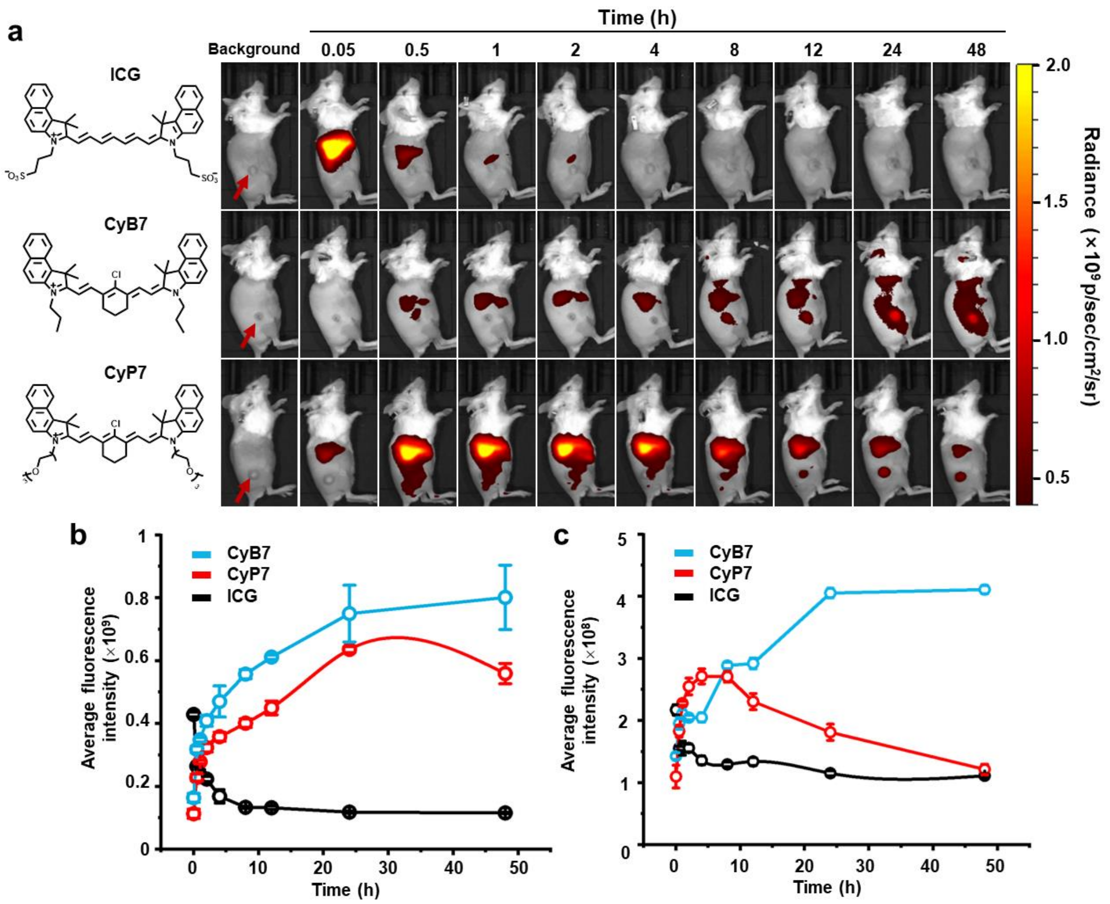

Supplementary Figure 38. (a) In vivo imaging of 4T1 tumor-bearing mice after intravenous injection of CyP7, CyB7 or ICG probes (5 nmol), the red arrows indicated tumor tissue. Fluorescence signal intensity in tumor tissue (b) and background (c) over time. Data are presented as mean ± SD. (n = 3 biologically independent mice per group)

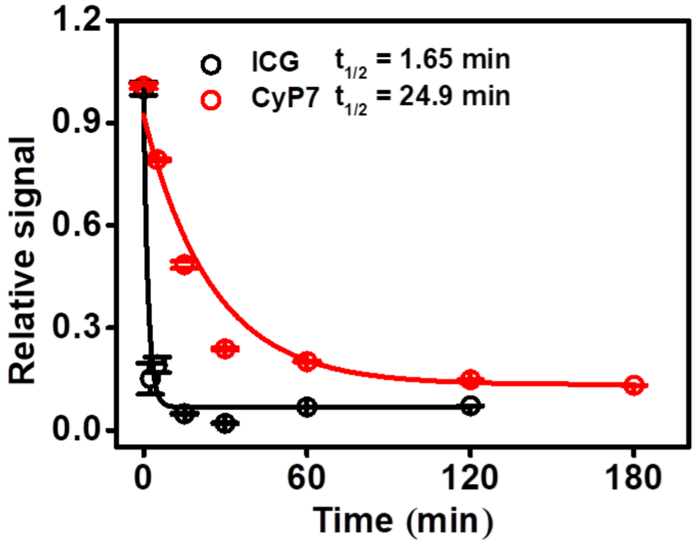

Supplementary Figure 39. Blood clearance curves of ICG and CyP7 from BALB/C mice by quantitative analyzing the fluorescence intensity of dyes in blood at different time points after intravenous injected with 50 nmol of each dye. Data are presented as mean ± SD. (n = 3 biologically independent mice per group).

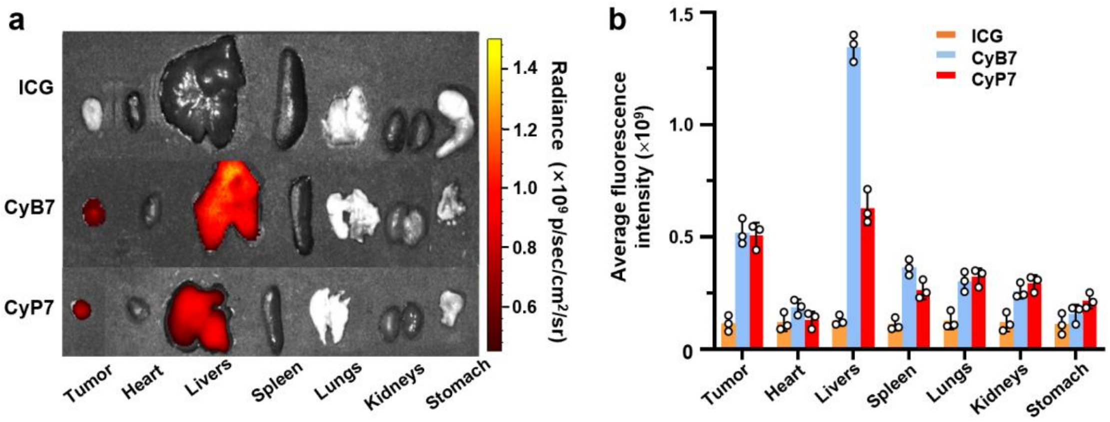

Supplementary Figure 40. Biodistribution analysis of CyP7, CyB7 and ICG probes treated mice group (5 nmol). Excised organs harvested from mice 24 HPI. (a) Ex vivo fluorescence images of major organs. (b) Fluorescence intensity analysis of anatomic organs. Data are presented as mean ± SD. (n = 3 biologically independent mice per group).

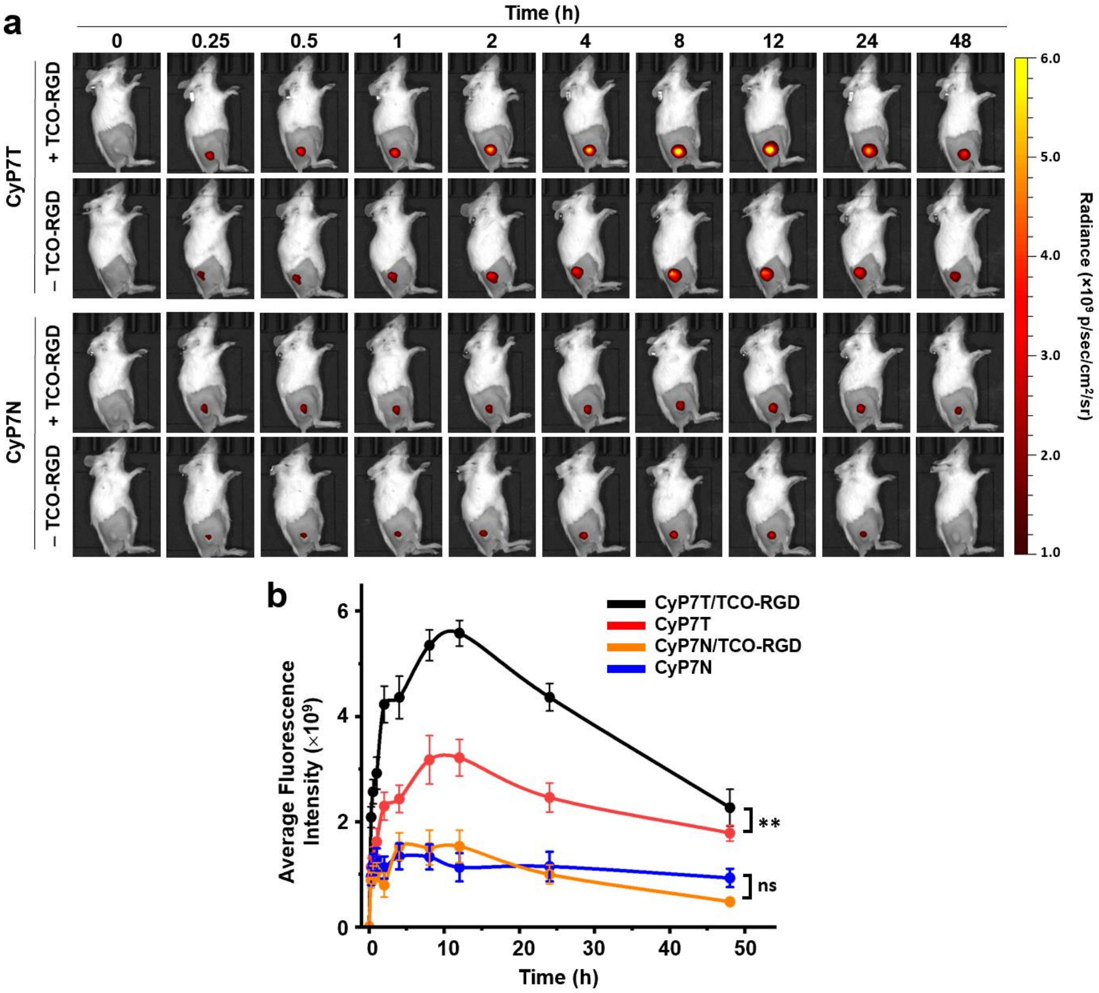

Supplementary Figure 41. In vivo imaging for 4T1 tumor-bearing mice after intratumor injection of probes. (a) NIR imaging was performed at various timepoints (0, 0.25, 0.5, 1, 2, 4, 8, 12, 24, 48 h) post-injection of CyP7T, CyP7T/TCO-RGD, CyP7N, or CyP7N/TCO-RGD probes (0.25 nmol). For bioorthogonal groups, mice were simultaneously injected with TCO-RGD (1 nmol) and CyP7T (0.25 nmol, or CyP7N). (b) Fluorescence signal intensity of tumor tissue in Supplementary Figure 41a over time. Data are presented as mean values ± SD from three separate measurements. A two-sided student's t-test was performed (CyP7T/TCO-RGD vs CyP7T, p = 0.0059; CyP7N/TCO-RGD vs CyP7N, p =0.8159). **: $p < 0.01$, ns: not significant.

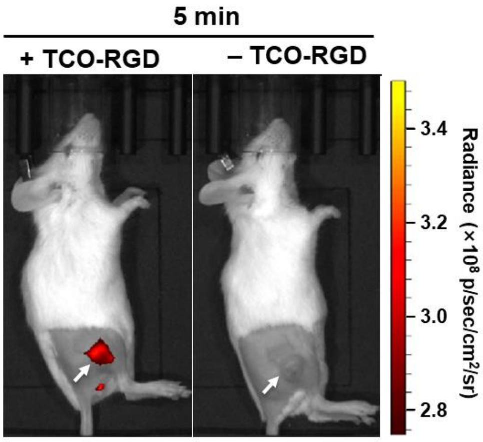

Supplementary Figure 42. In vivo imaging for 4T1 tumor-bearing mice after intravenous injection of CyP7T probes (5 nmol) at 5 min, the arrows indicated tumor tissue.

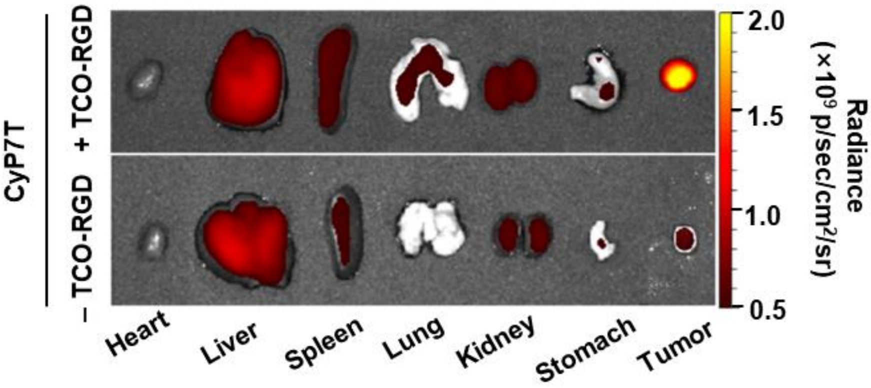

Supplementary Figure 43. Excised organs harvested from mice 24 HPI.

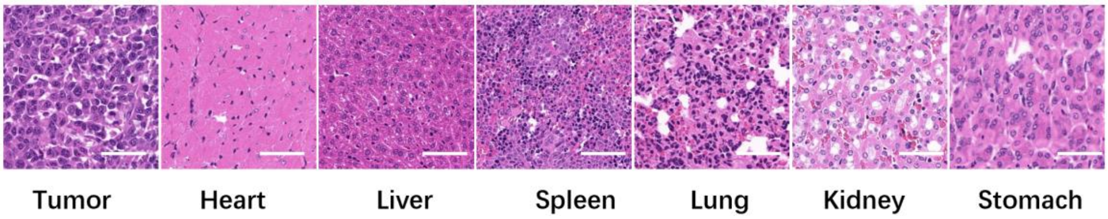

Supplementary Figure 44. Hematoxylin and eosin (H&E) pathology examinations for CyP7T/TCO. Scale bar: 50 μm. n = 3.

Supplementary Table 1. Optical properties of the cyanines.

Dye	λ_{abs} /nm	ε /$M^{-1}cm^{-1}$	λ_{em} /nm	Φ_F [a]	Stokes shift /nm
CyP7	821	253200	841	3.5%	20
CyP7-CH	679	216900	773	3.9%	94
CyP7T	679	204800	787	4.0%	108
CyP7TT	679	138500	786	18.6%	107
CyP7N	682	224320	794	5.6%	112
CyP7NT	686	244000	795	4.6%	109
CyP7N_3	672	121600	777	7.3%	105
CyP7N_3-BCN	671	135400	777	7.2%	106

Photophysical properties in methanol at 1 μM. [a] Fluorescence quantum yields of the dyes were determined by the reference standard (ICG Φ_F = 4.3% in methanol at room temperature).

Supplementary Table 2. Main calculated orbital transitions of the cyanines.

Dyes	Transition character	Transition proportion	Excitation energy ΔE	Oscillator Strengths f
CyP7	HOMO→LUMO	83.51%	1.9370 eV	2.6547
CyP7-CH	HOMO→LUMO	98.92%	2.0488 eV	1.8757
CyP7T	HOMO→LUMO	98.90%	2.0686 eV	1.8134
CyP7TT	HOMO-1→LUMO	97.47%	2.1173 eV	1.5209
CyP7N	HOMO-1→LUMO	99.18%	2.1111 eV	1.8340
CyP7NT	HOMO-1→LUMO	99.18%	2.1108 eV	1.8332
CyP7N_3	HOMO→LUMO	99.15%	2.1007 eV	1.8122
CyP7N_3-BCN	HOMO-1→LUMO	98.79%	2.0765 eV	1.7829

Supplementary References

1. Frisch, M. J. *et al.* Gaussian 09 (2009).

2. Berendsen H. J. C., van der Spoel D. & van Drunen R. GROMACS: A message-passing parallel molecular dynamics implementation. *Comput. Phys. Commun.* **91**, 43-56 (1995).

3. Dodda L. S., Cabeza de Vaca I., Tirado-Rives J. & Jorgensen W. L. LigParGen web server: an automatic OPLS-AA parameter generator for organic ligands. *Nucleic Acids Res* **45**, W331-W336 (2017).

4. Martínez L., Andrade R., Birgin E. G. & Martínez J. M. PACKMOL: A package for building initial configurations for molecular dynamics simulations. *J. Comput. Chem.* **30**, 2157-2164 (2009).

5. Humphrey W., Dalke A. & Schulten K. VMD: Visual molecular dynamics. *J. Mol. Graph.* **14**, 33-38 (1996).

6. Carlson, J. C. T., Mikula, H. & Weissleder, R. Unraveling tetrazine-triggered bioorthogonal elimination enables chemical tools for ultrafast release and universal cleavage. *J. Am. Chem. Soc.* **140**, 3603-3612 (2018).

7. Moore, J. W. & Pearson, R. G. Kinetics and mechanism. John Wiley & Sons (1981).

8. Dommerholt J., van Rooijen O., Borrmann A., Guerra C. F., Bickelhaupt F. M., van Delft F. L. Highly accelerated inverse electron-demand cycloaddition of electron-deficient azides with aliphatic cyclooctynes. *Nature communications* **5**, 5378 (2014).
