# Supplementary material for: Bioorthogonally activatable cyanine dye with torsion-induced disaggregation for in vivo tumor imaging
Source: Nat Commun. 2022 Jun 18;13:3513. doi: 10.1038/s41467-022-31136-3 (PMC9206667; doi:10.1038/s41467-022-31136-3)
Supplement: Supplementary file 2 — Reporting Summary [file 41467_2022_31136_MOESM2_ESM.pdf]

nature portfolio

Corresponding author(s): Jie Tian, Kanyi Pu, Zhongliang Wang

Last updated by author(s): Apr 23, 2022

Reporting Summary

Nature Portfolio wishes to improve the reproducibility of the work that we publish. This form provides structure for consistency and transparency in reporting. For further information on Nature Portfolio policies, see our Editorial Policies and the Editorial Policy Checklist.

Statistics

For all statistical analyses, confirm that the following items are present in the figure legend, table legend, main text, or Methods section.

n/a	Confirmed	
☐	☒	The exact sample size (n) for each experimental group/condition, given as a discrete number and unit of measurement
☐	☒	A statement on whether measurements were taken from distinct samples or whether the same sample was measured repeatedly
☐	☒	The statistical test(s) used AND whether they are one- or two-sided<br>*Only common tests should be described solely by name; describe more complex techniques in the Methods section.*
☒	☐	A description of all covariates tested
☒	☐	A description of any assumptions or corrections, such as tests of normality and adjustment for multiple comparisons
☐	☒	A full description of the statistical parameters including central tendency (e.g. means) or other basic estimates (e.g. regression coefficient) AND variation (e.g. standard deviation) or associated estimates of uncertainty (e.g. confidence intervals)
☐	☒	For null hypothesis testing, the test statistic (e.g. F, t, r) with confidence intervals, effect sizes, degrees of freedom and P value noted<br>*Give P values as exact values whenever suitable.*
☒	☐	For Bayesian analysis, information on the choice of priors and Markov chain Monte Carlo settings
☒	☐	For hierarchical and complex designs, identification of the appropriate level for tests and full reporting of outcomes
☒	☐	Estimates of effect sizes (e.g. Cohen's d, Pearson's r), indicating how they were calculated

Our web collection on statistics for biologists contains articles on many of the points above.

Software and code

Policy information about availability of computer code

Data collection	All the geometry optimization and molecular orbitals calculations were performed by the Gaussian 09 program package. Molecular dynamic simulations were performed by using Gromacs-2019.5 software. High-resolution mass spectra (HRMS) were performed on a Bruker OTOF-Q II mass spectrometer. 1H NMR spectra were recorded with a Varian 600 MHz NMR. The fluorescence spectra were measured in a Edinburgh Instruments' FLS1000. The imaging experiments in vitro were recorded on an Olympus FV 10i confocal fluorescent microscope. In vivo fluorescence imaging analysis was carried out in an IVIS Kinetic imaging system. FACS analysis was detected with a BD Accuri C6 flow cytometry.
Data analysis	Most of the statistical analyses were performed by Origin 8.0. VMD (version 1.9.4a51) and Open-Source PyMOL (version 2.5.0, Schrodinger, LLC.) software were used for trajectory surveillance and visualization. Qualitative analysis of cell confocal fluorescence were performed by ImageJ 1.52a.

For manuscripts utilizing custom algorithms or software that are central to the research but not yet described in published literature, software must be made available to editors and reviewers. We strongly encourage code deposition in a community repository (e.g. GitHub). See the Nature Portfolio guidelines for submitting code & software for further information.

Data

Policy information about availability of data

All manuscripts must include a data availability statement. This statement should provide the following information, where applicable:

- Accession codes, unique identifiers, or web links for publicly available datasets
- A description of any restrictions on data availability
- For clinical datasets or third party data, please ensure that the statement adheres to our policy

The data that support the findings of this study are available from the corresponding author upon reasonable request.

Field-specific reporting

Please select the one below that is the best fit for your research. If you are not sure, read the appropriate sections before making your selection.

[x] Life sciences   [ ] Behavioural & social sciences   [ ] Ecological, evolutionary & environmental sciences

For a reference copy of the document with all sections, see nature.com/documents/nr-reporting-summary-flat.pdf

Life sciences study design

All studies must disclose on these points even when the disclosure is negative.

Sample size	For in vivo imaging experiments, when the diameter of the mice tumor developed to be 4–6 mm after one-week inoculation, the mice were chosen for in vivo imaging. All sample sizes are listed in the corresponding figure legends or on the figures.
Data exclusions	No date were excluded from the analyses.
Replication	All experiments were conducted at least three times independently, and similar results were adopted for further analysis to guarantee reproducibility.
Randomization	For in vitro studies, the samples/cells were randomized into different groups prior to treatment. For in vivo experiments, the mice were randomly divided into different groups.
Blinding	Investigators were blinded to group allocation during cell and mice experiments.

Reporting for specific materials, systems and methods

We require information from authors about some types of materials, experimental systems and methods used in many studies. Here, indicate whether each material, system or method listed is relevant to your study. If you are not sure if a list item applies to your research, read the appropriate section before selecting a response.

Materials & experimental systems

n/a	Involved in the study	
[x]	[ ]	Antibodies
[x]	[ ]	Eukaryotic cell lines
[x]	[ ]	Palaeontology and archaeology
[ ]	[x]	Animals and other organisms
[x]	[ ]	Human research participants
[x]	[ ]	Clinical data
[ ]	[x]	Dual use research of concern

Methods

n/a	Involved in the study	
[x]	[ ]	ChIP-seq
[ ]	[x]	Flow cytometry
[x]	[ ]	MRI-based neuroimaging

Animals and other organisms

Policy information about studies involving animals; ARRIVE guidelines recommended for reporting animal research

Laboratory animals	6-8-week SPF female healthy BALB/c mice weighted 14-16 g were purchased from Chengdu Dossy Biological Technology Co. Ltd. The mice were housed in standard animal cages in the Laboratory Animal Center of Fourth Military Medical University.
Wild animals	No wild animals was used in this study.
Field-collected samples	No field collected samples were used in the study.
Ethics oversight	All animal operations in this article are in compliance with relevant regulations and ethical requirements. Animals received care in

accordance with the Guidance Suggestions for the Care and Use of Laboratory Animal Center of Fourth Military Medical University (20190226).

Note that full information on the approval of the study protocol must also be provided in the manuscript.

Dual use research of concern

Policy information about dual use research of concern

Hazards

Could the accidental, deliberate or reckless misuse of agents or technologies generated in the work, or the application of information presented in the manuscript, pose a threat to:

No	Yes	
☒	☐	Public health
☒	☐	National security
☒	☐	Crops and/or livestock
☒	☐	Ecosystems
☒	☐	Any other significant area

Experiments of concern

Does the work involve any of these experiments of concern:

No	Yes	
☒	☐	Demonstrate how to render a vaccine ineffective
☒	☐	Confer resistance to therapeutically useful antibiotics or antiviral agents
☒	☐	Enhance the virulence of a pathogen or render a nonpathogen virulent
☒	☐	Increase transmissibility of a pathogen
☒	☐	Alter the host range of a pathogen
☒	☐	Enable evasion of diagnostic/detection modalities
☒	☐	Enable the weaponization of a biological agent or toxin
☒	☐	Any other potentially harmful combination of experiments and agents

Flow Cytometry

Plots

Confirm that:

☒ The axis labels state the marker and fluorochrome used (e.g. CD4-FITC).

☒ The axis scales are clearly visible. Include numbers along axes only for bottom left plot of group (a 'group' is an analysis of identical markers).

☒ All plots are contour plots with outliers or pseudocolor plots.

☒ A numerical value for number of cells or percentage (with statistics) is provided.

Methodology

Sample preparation	4T1 cells were seeded on 24-well plates at the density of 1 × 10^5 cells per well, and cultured for 12 h to grow adherently. The cells were incubated with probes for 0.5, 1, 3 and 5 h, respectively. After culturing different time, then washed with PBS three times, the cells were digested, harvested, and quantitatively determined by BD Accuri C6 flow cytometry.
Instrument	BD Accuri C6 flow cytometry
Software	BD CFlow Plus
Cell population abundance	Around 10^5 cells were prepared for each sample, and 10^4 cells were analyzed by Flow cytometer.
Gating strategy	The gating strategy (FSC-A/SSC-A) was used to exclude cell debris and aggregates. The same gating strategy were applied to both control and experiment conditions.

☒ Tick this box to confirm that a figure exemplifying the gating strategy is provided in the Supplementary Information.
